# Integrating transcriptomics and metabolomics to analyze quinoa (*Chenopodium quinoa Willd.*) responses to drought stress and rewatering

**DOI:** 10.3389/fpls.2022.988861

**Published:** 2022-10-26

**Authors:** Xiuju Huan, Li Li, Yongjiang Liu, Zhiyou Kong, Yeju Liu, Qianchao Wang, Junna Liu, Ping Zhang, Yirui Guo, Peng Qin

**Affiliations:** ^1^College of Agronomy and Biotechnology, Yunnan Agricultural University, Kunming, China; ^2^College of Resources and Environment, Baoshan College, Baoshan, China; ^3^Graduate Office, Yunnan Agricultural University, Kunming, China

**Keywords:** quinoa, drought stress, transcriptomics, metabolomics, flavonoid biosynthesis

## Abstract

The crop production of quinoa (*Chenopodium quinoa Willd*.), the only plant meeting basic human nutritional requirements, is affected by drought stress. To better understand the drought tolerance mechanism of quinoa, we screened the drought-tolerant quinoa genotype “Dianli 129” and studied the seedling leaves of the drought-tolerant quinoa genotype after drought and rewatering treatments using transcriptomics and targeted metabolomics. Drought-treatment, drought control, rewatering-treated, and rewatered control were named as DR, DC, RW, and RC, respectively. Among four comparison groups, DC vs. DR, RC vs. RW, RW vs. DR, and RC vs. DC, we identified 10,292, 2,307, 12,368, and 3 differentially expressed genes (DEGs), and 215, 192, 132, and 19 differentially expressed metabolites (DEMs), respectively. A total of 38,670 genes and 142 pathways were annotated. The results of transcriptome and metabolome association analysis showed that gene-*LOC110713661* and gene-*LOC110738152* may be the key genes for drought tolerance in quinoa. Some metabolites accumulated in quinoa leaves in response to drought stress, and the plants recovered after rewatering. DEGs and DEMs participate in starch and sucrose metabolism and flavonoid biosynthesis, which are vital for improving drought tolerance in quinoa. Drought tolerance of quinoa was correlated with gene expression differences, metabolite accumulation and good recovery after rewatering. These findings improve our understanding of drought and rewatering responses in quinoa and have implications for the breeding of new drought-tolerance varieties while providing a theoretical basis for drought-tolerance varieties identification.

## Introduction

Quinoa (*Chenopodium quinoa* Willd.) is native to the Andean region of South America, with a long history of cultivation and has received considerable attention as a functional food in recent years. Quinoa grains are small and round with white, black, and red colors (Repo-Carrasco et al., 2003). Their outstanding physicochemical, nutritional, and functional properties result in their use as a staple food for humans (Melini and Melini, 2021). With its high nutritional potential and genetic diversity, the Food and Agriculture Organization (FAO) classified quinoa as a promising crop for humans that can contribute to food security in the 21st century (FAO Regional Office for Latin America and the Caribbean & Proinpa, 2011). Therefore, the selection and exploitation of quinoa varieties is crucial.

Quinoa contains high quality protein with a perfect balance of essential amino acids and a suitable fatty acid composition. In addition, it is rich in bioactive compounds such as polyphenols, flavonoids, and minerals (Nowak et al., 2016); the high content of phenolics makes it an important antioxidant active. The polysaccharide fraction of quinoa also has potential as a natural antioxidant, antidiabetic, and immunomodulatory food (Tan et al., 2021). Both the seeds and leaves of quinoa have some nutritional value, and current research on quinoa has focused on the nutritional composition of the seeds as functional foods. However, quinoa leaves also have some nutritional potential and may prevent cancer and other diseases related to oxidative stress (Gawlik-Dziki et al., 2013). Quinoa grains and leaves both contain a high content of phenolics, although the leaves have a higher protein content than the grains. The consumption of nutrient-rich green leaf quinoa can prevent nutritional deficiencies caused by iron and zinc, and there is higher saponin content in the grains compared with the leaves (Pathan et al., 2019; Villacrés et al., 2022). Quinoa grains also contain sufficient micronutrients such as calcium, phosphorus, potassium, copper, iron, and zinc (Ayasan, 2020). In addition, quinoa has some anti-nutritional factors such as saponins, phytic acid, tannins, and protease inhibitors, among which the saponins can resist the adverse conditions of the external environment (Filho et al., 2017). Quinoa has a wide genetic diversity and can adapt to various harsh environments including biotic and abiotic stresses, and can be grown on plateaus from sea level to 4,500 m (Zhang et al., 2021). However, the nutritional value of quinoa differs by variety and environment (Nowak et al., 2016), making it particularly important to study quinoa breeding in adverse conditions.

Drought conditions threaten crop production and food security (Yang et al., 2020). Drought stress affects morphological and physiological changes in plants, leading to severe crop yield deficits; agricultural drought affects global food production and is among the most serious challenges facing sustainable agriculture (Fadiji et al., 2022). Water stress not only affects metabolic activities such as plant respiration, sugar metabolism, and photosynthesis (Dos Reis et al., 2012), but also reduces the cellular water potential, affecting growth and cell elongation. The occurrence of drought stress during the reproductive period may also lead to interruption of flowering and yield loss (Kaur and Asthir, 2017). Plants under drought stress release reactive oxygen species (ROS) and free radicals, triggering an increase in ethylene content (Nair et al., 2008; Narayanasamy et al., 2020). Drought stress not only causes physiological responses in plants, but also impacts mineral nutrition, where a reduction in iron uptake occurs through a specific response to drought, leading to a reduction in zinc and manganese uptake, which is associated with differential expression of transport-related genes (D'Oria et al., 2022). Drought tolerance in plants refers to the ability of plants to tolerate drought and rapidly resume growth after rehydration. Drought severely affects plant growth and development; however, there is some compensation through rehydration (e.g., photosynthesis) (Chaves and Oliveira, 2004). In conclusion, increasing future food production under drought stress will be challenging. While many crops have been extensively studied under drought stress, research on the unique mechanisms of quinoa, an important gluten-free crop, to cope with different degrees of drought have been limited. Among the mechanisms of action for drought adaptation in quinoa, bio-promoters can lead to an increase in total soluble sugars (SS), proteins, and antioxidant enzyme activities in quinoa leaves and roots; however, drought decreases biomass, leaf water potential, and stomatal conductance, and increases malondialdehyde and hydrogen peroxide content (Benaffari et al., 2022). Physiological analysis of drought tolerance mechanisms in quinoa demonstrated an increase in H_2_O_2_ and malondialdehyde (MDA) content in drought-treated quinoa, with differences in the physiological response to different varieties of quinoa. This suggests that different varieties of quinoa have different drought tolerance mechanisms (Lin and Chao, 2021). Further, physiological characteristics of quinoa under rehydrated conditions after drought stress have not been reported. Quinoa genotypes grown in coastal lowlands have always exhibited better yields and larger seeds with reduced irrigation relative to commercial varieties (Dumschott et al., 2022). Among them, *CqZF-HD14* further enhanced the drought tolerance of quinoa seedlings in synergy with *CqNAC79* or *CqHIPP34* and may be a key gene in the drought tolerance regulatory network of quinoa (Sun et al., 2022). Varieties and methods that can withstand drought stress, among others, are being extensively researched worldwide to alleviate water stress (Philippot et al., 2013). Thus, it is imperative to investigate the mechanisms of drought tolerance in quinoa in order to successfully breed drought-tolerant varieties.

Transcriptome sequencing technologies and metabolome assays have been used to analyze plant tolerance mechanisms (Lenka et al., 2011; Nakabayashi et al., 2014; Yuan et al., 2018; Mu et al., 2021). Comparative transcriptome analysis of drought tolerance in two rice varieties showed that it was attributable to enhanced expression of several enzyme-coding genes, and drought sensitivity was attributed to significant down-regulation of regulatory components that confer drought tolerance (Lenka et al., 2011). Ramie plants exhibited differential expression of *AP2, MYB, NAC*, zinc finger proteins, and the *bZIP* transcription factor (TF) (An et al., 2015), suggesting an association with osmotic treatment. The maximum activity of superoxide dismutase (SOD) and peroxidase (POD), as well as the contents of MDA and proline (Pro), increased in wheat plants to differing degrees during a winter drought (Mu et al., 2021). Flavonoids can alleviate oxidation and drought stress in *Arabidopsis thaliana* (Nakabayashi et al., 2014). In the study of Tibetan hullless barley under salt stress the main compounds included amino acids and their derivatives, organic acids, nucleotides and their derivatives, and flavonoids (Wang et al., 2019). Transcriptomic and metabolomic studies on powdery mildew tolerance in Tibetan hullless barley showed a significant enrichment of genes related to pathways such as phenylalanine metabolism, terpene biosynthesis, zeatin biosynthesis, and isoflavonoid biosynthesis, which may be associated with downy mildew tolerance in fully tolerant varieties (Yuan et al., 2018).However, a multi-omics-based study of drought tolerance mechanisms in quinoa seedlings has not yet been reported.

Therefore, it is imperative to rapidly screen genes associated with drought stress and rehydration to provide excellent genetic resources for creating drought-tolerant quinoa germplasm. However, studies on the molecular regulation of quinoa adaptation to drought stress have been limited, and multi-omics-based studies on drought tolerance mechanisms in quinoa seedlings have not yet been reported. In this study, we analyzed drought-tolerant quinoa plants at the seedling stage (six-leaf stage) after drought and rewatering by transcriptome sequencing and metabolomics. Our results shed light on the mechanisms of drought tolerance in quinoa and direct research towards a comprehensive exploration of drought tolerance genes in quinoa. In addition, this topic is important to understand the drought tolerance mechanism of quinoa in order to breed optimal varieties.

## Materials and methods

### Material planting

Material was sourced from China and the United States, and quinoa genotypes were introduced through selective breeding (The natural variation of existing varieties during the breeding process is used as the original material for the selection work, which is then carried out continuously according to the requirements of high and stable yield and disease resistance, and the selected lines are planted and selected again until the traits are relatively stable). While quinoa grains are available in a variety of colors, four colors were initially selected for this study: red, yellow, white, and black, with five genotypes of each color for a total of 20 an advance generation genotypes. The 20 quinoa genotypes were planted in a greenhouse seedling tray. Fifty-cell seedling growth trays were used (50 mm × 50 mm × 90 mm for each point). Three seeds were sown at each point, the seedlings were thinned when the plants had grown to the two-leaf stage, and finally, one plant was left at each point. All genotypes were managed in the same way with water and fertilizer before the drought treatment. Humus soil was mixed with perlite at a 4:1 ratio as the cultivation substrates for the drought stress-treated and control groups.

### Drought treatment and drought-tolerance material screening

Quinoa seedlings were treated with natural drought stress (i.e., no watering during this period) for 5 ds at the 6-leaf-one stage, followed by 1 day of rewatering. The drought-treated control was watered normally during the drought treatment and the rewatered control was watered normally on both the 5 days of the drought treatment and the 1 day of rewatering. The average temperature of the greenhouse was 27.1°C and the average humidity was 59.6%. Based on the degree of wilting and survival rate of plants on the fifth day of drought treatment, two drought tolerant genotypes (Dianli 66 and Dianli 129) and two drought sensitive genotypes (Dianli 58 and Dianli 114) were initially screened out of 20 genotypes. Only a few plants in each genotype with slightly curled leaves were considered drought tolerant and most plants in each genotype with all curled leaves was considered drought sensitive, according to degree of wilting criteria used. For the survival rate criteria, seedlings of quinoa genotypes with more than 85% survival are considered drought tolerant genotypes and those with less than 60% survival are considered drought sensitive genotypes. The most drought tolerant genotype, Dianli 129, and the most drought sensitive genotype, Dianli 114, were further screened among the four genotypes according to the method above. The leaves of these two genotypes were sampled on day 5 of the drought treatment and on day 1 of the rewatering treatment, with DR on day 5 of the drought treatment, drought treatment was rewatered for 1 day after 5 days as RW and normal watering during both the drought and rewatering treatments was the rewatering control RC. The physiological parameters related to drought tolerance were measured, and the drought tolerant genotypes and drought sensitive genotypes were analyzed for comparison (Lin and Chao, 2021). Finally, metabolome determination and transcriptome sequencing were performed on the most drought tolerant genotype, Dianli 129, to further investigate the drought tolerance mechanism of quinoa. Dianli 129 had four treatments, drought treatment (drought treatment for 5 days), drought control (normal watering), rewatering treatment (drought treatment for 5 days followed by rewatering for 1 day) and rewatering control (normal watering), each with three replicates for a total of 12 samples, one sample from each treatment was mixed with five biological replicates.

### Morphological parameters, physiological parameters measurement and statistical analysis

The most drought tolerant genotype, Dianli 129, and the drought sensitive genotype, Dianli 114, were screened for morphological and physiological parameters measurement. Single quinoa seedlings of uniform growth were selected to determine plant height, above- and below-ground part biomass, and leaf morphology and each parameter were replicated three times. The root length, average root diameter, volume and root surface area were determined by scanning with a Topper root scanner (MRS-9600TFU2L). The plants were placed in an oven, killed at 110°C for 15 min, dried at 80°C until a constant weight, and the dry weight of above- and below-ground parts was measured, and the root-to-crown ratio = root dry weight/above-ground part dry weight was calculated. Leaf color was measured by a Minolta colorimeter (CR-20). Among the physiological parameters determined, Chlorophyll content was determined using the ethanol acetone method. Soluble protein content was determined by the Komas blue colorimetric method. Pro content was determined by the acidic ninhydrin method. MDA content was determined by the thiobarbituric acid (TBA) method. Catalase (CAT) activity was determined by the hydrogen peroxide reduction method. POD activity was determined by the guaiacol method. SOD was determined by the nitrogen blue tetrazolium photochemical reduction method. Total antioxidant capacity (T-AOC) was determined by the iron ion reduction method. SS were determined by the anthrone colorimetric method. Relative conductivity was also measured. Each parameter was repeated three times (Benaffari et al., 2022). Microsoft Excel 2010 was employed for graphical analysis and statistical analysis was performed using SPSS25, DPS version 7.05 software.

### Metabolite extraction and detection

The samples were placed in a SCIENTZ-100F Lyophilization Dryer Laboratory LCD Display Freeze Dryer (SCIENTZ) for vacuum freeze-drying. The samples were ground to powder form using a MM 400 grinding machine (30 Hz, 1.5 min; Retsch). Subsequently, 100 mg of powder was dissolved in a 1.2 mL 70% methanol extract. The dissolved samples were refrigerated overnight at 4°C, while swirling six times during the period to improve extraction rate. Each sample was centrifuged at 12,000 rotations per min for 10 min, after which the upper liquid fraction was filtered through a 0.22-μm membrane filter (0.22-μm pore size). Each sample was saved in a bottle for analysis using ultra-performance liquid chromatography and tandem mass spectrometry.

Metabolome profiling was performed using a widely targeting metabolomics method, based on a database (MWDB) built by Wuhan Mettware Biotechnology (http://www.metware.cn/), and qualitative analysis was based on secondary spectrum information. Metabolites were quantitated using triple-level quadrupole mass spectra obtained in multiple-reaction monitoring mode. Before data analysis, quality-control analysis was performed to confirm the reliability of the data. Principal component analysis (PCA) was conducted to analyze variabilities between and within groups. DEMs were subjected to orthogonal partial least-squares discriminant analysis (OPLS-DA). Metabolites with a variable importance in projection (VIP) ≥ 1 and a fold-change (FC) of ≥ 2 (or ≤ 0.5) were defined as DEMs. See **Supplementary Material 1** for the collection conditions and experimental methods of chromatography-mass spectrometry.

### Transcriptome sequencing and data analysis

Total RNA was extracted from 12 quinoa leaf samples using the TRIzol reagent according to the manufacturer’s instructions (Beijing TransGen Biotech). After RNA extraction, an RNA-sequencing library was constructed, and then the quality of the library was determined. The Illumina HiSeq platform was used for sequencing after determining that the sequencing library met the requirements. To ensure the accuracy of subsequent analysis, the original data were filtered and screened, and low-quality reads, adapter sequences, and when the N content (proportion of reads with N bases) of any sequenced read exceeds 10% of the number of bases in that read were removed. The high-quality clean reads obtained by screening were compared with the reference genome. The fragments per kilobase of transcript (FPKM) per million mapped reads was used as an parameters to measure gene expression levels. The screening criteria for identifying DEGs were a |log2 FC| value of ≥ 1 and a false-discovery rate of < 0.05, a positive value of |log2 FC| is an up-regulated gene, while a negative value is a down-regulated gene. Functional annotations of DEGs were performed using the Kyoto Encyclopedia of Genes and Genomes (KEGG), Gene Ontology (GO), Eukaryotic Orthologous Group (KOG), PfAM, Swiss-Prot, TrEMBL, and NR databases. See **Supplementary Material 2** for the experimental procedure of transcriptome sequencing.

### Quantitative real-time PCR validation (qRT-PCR)

qRT-PCR was conducted in Step One in addition to a real-time fluorescence quantitative PCR instrument (Thermo Fisher, USA). TUB1 was used as the internal reference gene. The reaction procedure was as follows: 95°C for 30 s, followed by 40 cycles, 95°C for 5 s, and 60°C for 30 s. According to the kit instructions (Beijing TransGen Biotech), a 20 μL system was used for each reaction: 3 μL cDNA, 0.4 μL forward primer (10 μM) and 0.4 μL reverse primer (10 μM), 10 μL green qPCR SuperMix, 0.4 μL passive reference dye I, and 5.8 μL nuclease-free water. The experiment was repeated with three biological replicates on 96-well plates.

## Results

### Effects of drought stress and rewatering on the morphology and physiology of quinoa seedlings

The morphological parameters (total root length, total root surface area, total root volume, etc.) and physiological parameters (CAT, SOD, MDA, T-AOC, etc.) of the drought tolerant genotype Dianli-129 and drought-sensitive genotype Dianli 129 were compared. The difference of morphological indexes between two strains “dianli-114” and “dianli-129” was compared. The root shoot ratio was P > 0.05, so the difference was not significant. The total root length and other six indexes were P<0.01, so the difference was extremely significant; The comparison among the four treatments (DR, DC, RW, RC) showed that the root shoot ratio was P < 0.05, so the difference was significant. The leaf area and other six parameters were P < 0.01, so the difference was extremely significant (**Table 1**). Compared between the two strains, leaf width P < 0.05, the difference is not significant, and leaf perimeter and other seven parameters P <0.01, the difference is extremely significant; Compared among the four treatments, all indexes were P < 0.01, the difference was extremely significant (**Table 2**). The ANOVA found no significant differences in CAT, SOD, MDA and T-AOC between the two genotypes, however, significant differences in PDO and all other parameters were identified. In each treatment, the difference of all parameters was highly significant; Under the cross action of the two factors, the chlorophyll difference was significant, and all other parameters reached a highly significant level (**Table 3**).

**Table 1:** Analysis of variance for morphological parameter determination 1 (*F* value).

Source of variation	DF	Total root length (cm)	Total root Surface Area (cm^2^)	Total root volume (cm^3^)	Average root Diameter (mm)	Plant height(cm)	Leaf area(mm^2^)	Root-shoot ratio
Materials	1	150.409**	374.858**	559.578**	29.356**	757.412**	11.689**	1.850
Treatments	3	112.004**	269.226**	274.101**	13.582**	249.828**	203.869**	6.319*
Materials×Treatments	3	102.450**	231.390**	407.149**	8.466**	255.951**	20.927**	19.002**

* and ** indicate P<0.05 and P<0.01. **Table 2**.

**Table 2:** Analysis of variance for morphological parameter determination 2 (*F* value).

Source of variation	DF	Root dry weight	Shoot dry weight	Leaf length	Leaf width	Leaf perimeter	Lightness L	Red-Green a	Yellow-Blue b
Materials	1	16.0**	15.4**	51.5**	2.5	14.5**	21.9**	2040.0**	19.4**
Treatments	3	31.2**	11.8**	45.1**	172.0**	41.6**	74.2**	874.7**	1155.0**
Materials×Treatments	3	50.8**	15.6**	22.2**	10.7**	14.7**	0.9	500.1**	119.6**

* and ** indicate P<0.05 and P<0.01. **Table 3**.

**Table 3:** Analysis of variance for physiological parameter determination(*F* value).

Source of variation	DF	CAT	POD	SOD	MDA	Proline	T-AOC	Soluble protein	Soluble sugar	Relative conductivity	Chlorophyll
Materials	1	1.8	6.6*	1.14	0.22	94.2**	0.74	238**	29.7**	46.2**	23.183**
Treatments	3	140**	71**	79.0**	65**	1885**	26.5**	133**	199.9**	43.6**	8.154**
Materials×Treatments	3	272**	218**	55.1**	21**	278**	9.73**	132**	28.8**	25.3**	3.747*

* and ** indicate P < 0.05 and P < 0.01, respectively. DF, degree of freedom; CAT, catalase; POD, Peroxidase; MDA, Malondialdehyde; T-AOC, Total antioxidant capacity.

### Metabolomics of quinoa leaves under drought and rewatered conditions

**Figure 1:**
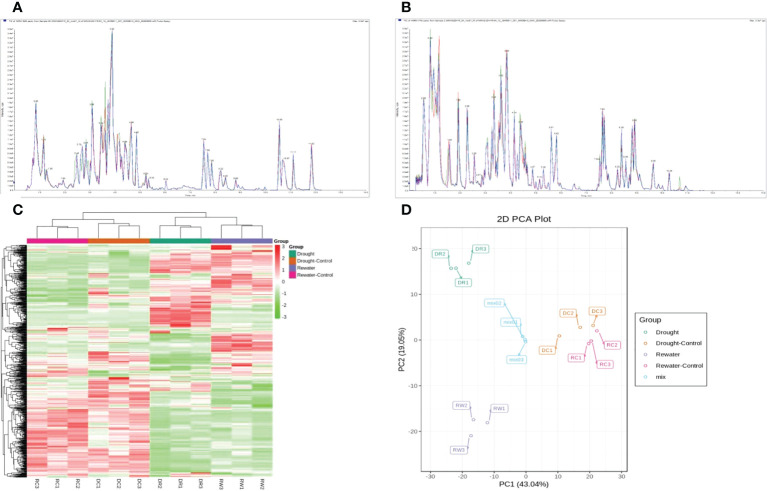
Analysis of metabolites in different comparison groups. **(A, B)** TIC overlap diagram of indicating the sample qualities, based on the observed spectra. **(C)** Overall clustering diagram for several samples. **(D)** PCA diagram. In **(A, B)**, **(A)** indicates the for negative-ion mode, while **(B)** indicates the positive-ion mode. In **(C)**, the sample names are shown horizontally, the metabolite information is shown vertically, and the values obtained after standardizing the relative contents are shown in different colors (red represents a high content, green represents a low content). In **(D)**, PC1 represents the first principal component, PC2 represents the second principal component, and the percentages represent the estimated contribution of the principal component to the data set. Each data point in the figure represents a sample. Samples in the same group are represented using the same color, and “MIX” is the quality-control sample containing a mixture of equal amounts of each sample.

Four groups of samples (DR, DC, RW, and RC) were analyzed using a widely targeted metabolomics approach that enabled the detection of 701 metabolites divided into 12 categories including amino acids and derivatives, flavonoids, and phenolic acids. A total of 99 flavonoids related metabolites were detected, namely 53 flavonols, 36 flavonoids, 4 dihydroflavonols, 2 dihydroflavone, 2 isoflavones, and 2 chalcones. The contents of 11 flavonoids such as 6-hydroxyluteolin 5-glucoside were lower than the control during drought but increased to the control level or significantly higher after rehydration. The content of 15 flavonoids such as kaempferol-4’-o-glucoside increased during drought, and the content after rehydration exhibited little difference compared with the control or continued to increase after rehydration. The contents of 58 flavonoids such as naringenin (5,7,4’-trihydroxyflavanone) were significantly lower than the control during drought. After rehydration, their contents did not adjust to the control level; however, they all exhibited varying upward trends (**Supplementary Table 1**). The total ion current (TIC) diagram of total ion flow shows detection and analysis of essential spectra for different quality-control samples, which overlapped. The total ion flow metabolite curves showed a high degree of overlap, that is, the retention times and peak strengths were consistent. This indicates that the signal stability of mass spectrometry for detecting the same sample at different time points was good, that is, the technology of metabolite extraction and detection had good repeatability and high reliability (**Figures 1A, B**). Cluster-heat map analysis was performed on all samples. All samples grouped together after performing three replicates, indicating that the metabolome data had high reliability. Significant differences were found between the control groups (RC and DC) and the treatment groups (DR and RW), in terms of the metabolite levels. After rewatering treatment, the metabolites slowly returned to normal levels After rewatering treatment, the metabolites slowly returned to normal levels (**Figure 1C**). PCA of the samples revealed that there was an evident trend of separation between groups on PC1 and PC2, differences among the groups and good repeatability (**Figure 1D**).

### Identification of different metabolites in quinoa leaves

**Figure 2:**
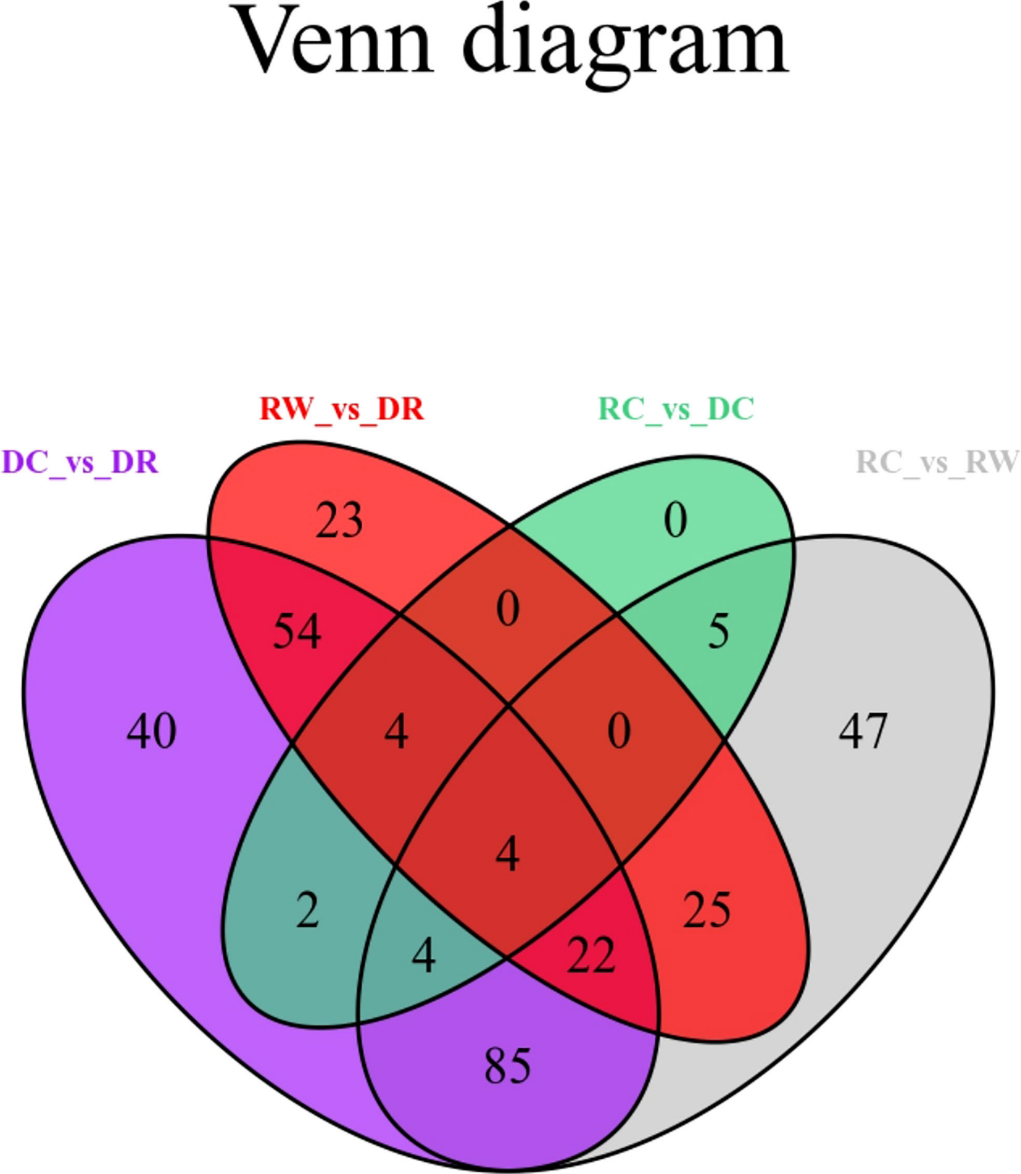
Venn diagram for differential metabolites. Each circle in the figure represents a comparison group. The numbers in the circles and overlapping regions represent the number of differential metabolites in common with the comparison group, whereas the numbers without overlaps represents the number of DEMs unique to the comparison group.

**Figure 3:**
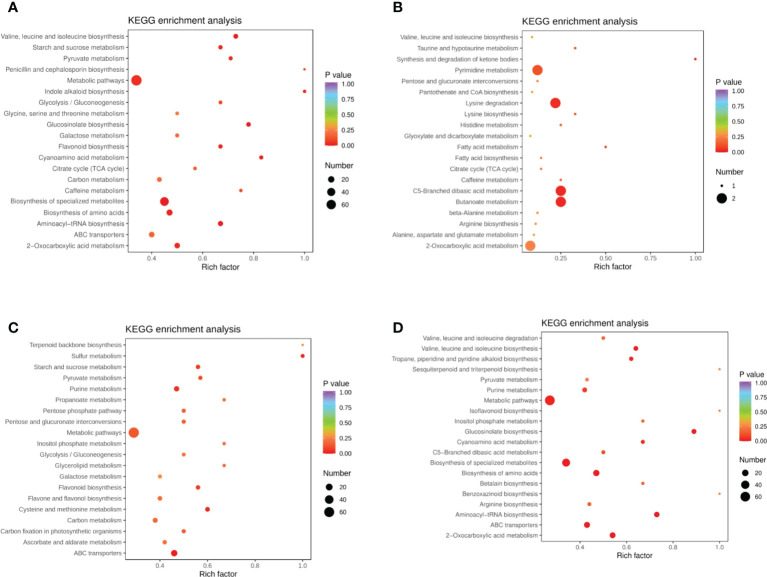
KEGG enrichment diagram of different metabolites in quinoa leaves. **(A)** DC vs DR. **(B)** RC vs DC. **(C)** RC vs RW. **(D)** RW vs DR. The horizontal coordinate represents the Rich factor corresponding to each pathway (i.e., the ratio between the number of metabolites in the corresponding pathway and the total number of metabolites detected and annotated in the pathway, where larger Rich factors correspond to greater enrichment). The vertical coordinate represents pathway name, the color of each data point represents the P value (where a deeper shade of red corresponds to more significant enrichment). The size of each data point represents the number of enriched DEMs.

DEMs were identified between samples according to the criteria of a VIP of ≥1 and an FC of ≥ 2 or ≤ 0.5. To study trends in different samples, the relative contents of different metabolites were standardized, centralized, and analyzed by K-means clustering (**Supplementary Figure 1**; **Supplementary Table 2**). The different metabolites were divided into nine groups, In the 6th cluster, the levels of amino acids and their derivatives with higher metabolite levels under drought conditions, which returned to normal after rewatering. In the seventh cluster, metabolites such as flavonoids were lower under drought conditions and returned to normal after rewatering. Four differential metabolite-comparison groups were obtained through pairwise comparisons. Specifically, in the DC vs DR comparison group, 84 metabolites were up-regulated and 131 were down-regulated. In the RC vs RW comparison group, 84 metabolites were up-regulated and 108 were down-regulated. In the RW vs DR comparison group, 67 metabolites were up-regulated and 65 were down-regulated. In the RC vs DC comparison group, 15 metabolites were up-regulated and four were down-regulated (**Supplementary Table 3**). The Venn diagram in **Figure 2** shows that the different groups had 40, 23, 0, and 47 DEMs, respectively, four of which were common among all four groups. By comparing the metabolite FCs in each group, we determined that among the four groups, up-regulated DEMs with the largest |log2 FC| values included N-feruloyltyramine, quercetin-7-O-rutinoside-4-O-glucoside,4-O-(6-O-glucosylferuloyl)-3,4-dihydroxybenzyl alcohol, and quercetin-7-O-rutinoside-4-O-glucoside. Down-regulated DEMs with the highest |log2FC| values included quercetin, 4-O-(6-O-glucosylferuloyl)-3,4-dihydroxybenzyl alcohol, quercetin, and LysoPE 15:1 (**Supplementary Figure 2)**. In the four differential metabolite-comparison groups, the DEM-associated metabolite pathways with significant enrichment included cyanoamino acid metabolism, flavonoid biosynthesis, starch and sucrose metabolism, penicillin and cephalosporin biosynthesis, indole alkaloid biosynthesis, sulfur metabolism, propanoate metabolism, glycerolipid metabolism, glucosinolate biosynthesis, aminoacyl-tRNA biosynthesis, synthesis and degradation of ketone bodies, fatty acid metabolism, and lysine biosynthesis (**Figure 3**; **Supplementary Figure 3**).

### Transcriptomics of quinoa leaves under drought and rewatered conditions

**Figure 4:**
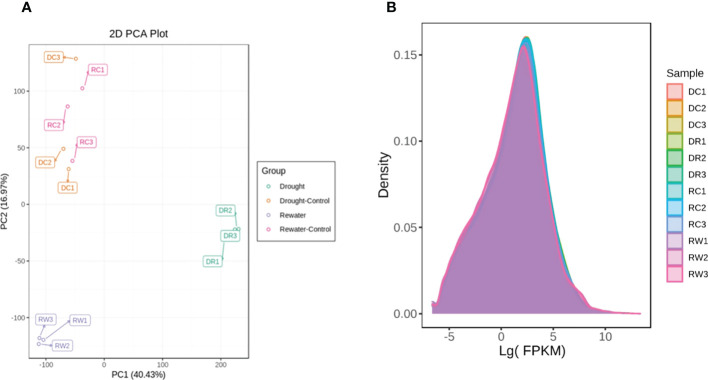
PCA of the different comparison groups analyzed in this study. **(A)** PCA diagram. **(B)** Diagram showing the expression-density distribution of PC1 in panel A representing the most obvious features that could be described in the multidimensional data matrix. The distribution for PC2 represents the most significant features that could be described in the data matrix, except for PC1. The curves are indicated with different colors to represent different samples. The abscissa represents the logarithm of FPKM of each sample and the ordinate represents the probability density.

Transcriptome sequencing analysis of 12 samples yielded 77.06 GB clean data, Among the high-quality clean reads, the percentage of the Q20 base was >98%, the percentage of the Q30 base was >94%, and the GC contents were >43.0%. These reference data indicated that the sequencing results were reliable and could be used for further analysis (**Supplementary Table 4**). By adopting the PCA method of multivariate statistical analysis, the data for each group of triplicate samples showed that the method had good stability and quality. Significant separation was found between the treated and control samples, indicating that changes in metabolite accumulation were strictly controlled by differential gene expression (**Figure 4A**). Using FPKM as an parameter of gene expression levels, the density map showed that the gene-abundance trends in the samples changed with the expression levels, which clearly reflected the gene expression levels in the samples (**Figure 4B**), FPKM = 10^-2^ ~ 10^4^. Pearson’s correlation coefficient (PCC, abbreviated as “r”) was used as an parameter to evaluate correlations with biological replicates. The closer the R2 is to 1, the stronger the correlation between the two replicate samples. This study requires that the R2 between biological replicate samples be at least greater than 0.8 before further study of DEGs.

### DEGs in quinoa leaves

**Figure 5:**
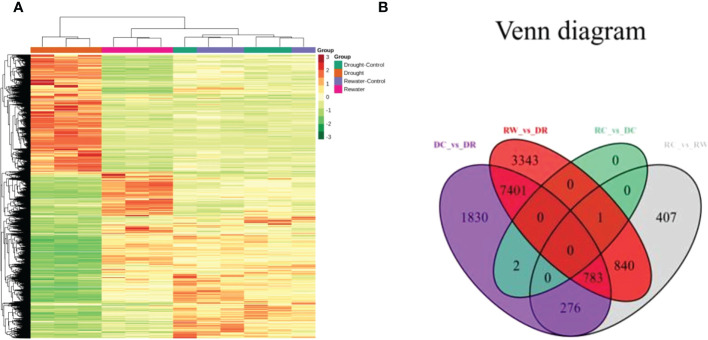
Cluster analysis of gene-expression data and a Venn diagram related to DEGs. **(A)** The abscissa represents the sample names and hierarchical clustering results, whereas the ordinate represents DEGs and hierarchical clustering results. Red shading indicates high expression, and green shading indicates low expression. **(B)** The non-overlapping regions represent specific DEGs for each group, and the overlapping areas represent DEGs common to the indicated subgroups.

**Figure 6:**
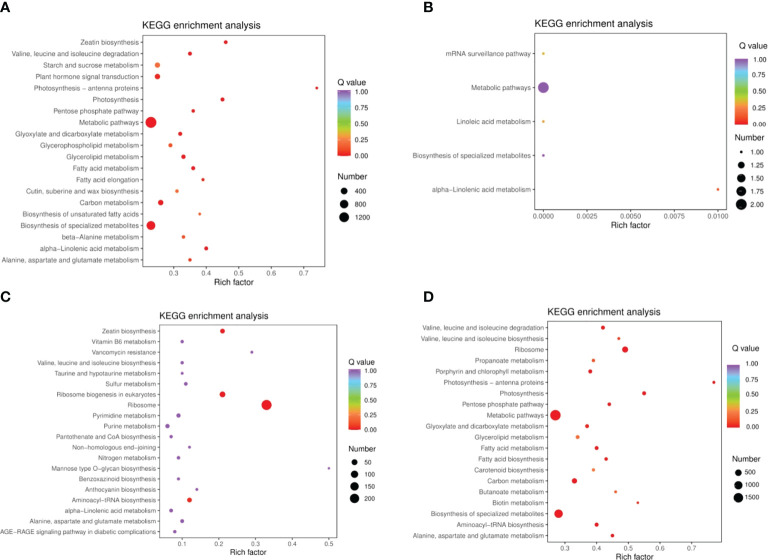
KEGG enrichment analysis of DEGs. **(A)** DC vs DR **(B)** RC vs DC **(C)** RC vs RW **(D)** RW vs DR. The ordinate represents the KEGG pathway. The abscissa represents the Rich factor the ratio of the number of different genes enriched in a pathway to the total number of annotated genes, where greater Rich factors correspond to greater enrichment. The larger the data point, the greater the number of DEGs enriched in the pathway. Deeper red shading indicates greater statistical significance in terms of the enrichment.

Using the following databases, we annotated of DEGs with KEGG (38,670; **Supplementary Table 5**), GO (38,191; **Supplementary Table 6**), KOG (45,935; **Supplementary Table 7**), PfAM (77,193; **Supplementary Table 8**), Swiss-Prot (31,837; **Supplementary Table 9**), TrEMBL (47,387; **Supplementary Table 10**), and NR (49,054; **Supplementary Table 11**). KEGG involves 142 pathways. By analyzing DEGs in quinoa leaves under drought stress and rewatering conditions, 14,883 differentially expressed genes were found. When comparing them in pairs, we generated four DEG comparison groups, among which 4,104 up-regulated genes and 6,188 down-regulated genes were found in the DC vs. DR comparison group. In the RC vs. RW comparison group, there were 1,439 up-regulated genes and 868 down-regulated genes. In the RW vs. DR comparison group, there were 5,374 up-regulated genes and 6,994 down-regulated genes. In the RC vs. DC comparison group, there were no up-regulated genes and three down-regulated genes (**Supplementary Table 12**). After drought treatment, the gene expression patterns changed significantly, and the genes tended to be stably expressed after rewatering (**Figure 5A**). The Venn diagram in Figure 5B shows 0 DEGs in common among all four groups, and 1,830, 3,343, 0, and 407 specific DEGs in the four comparison groups, respectively. KEGG analysis (**Figure 6** and **Supplementary Figure 4**) showed that the significantly enriched pathways in the four comparison groups included zeatin biosynthesis, photosynthesis, photosynthesis-antenna proteins, ribosome, ribosome biogenesis in eukaryotes, biotin metabolism, alpha-linolenic acid metabolism, and linolenic acid metabolism. The DEGs in quinoa leaves were classified by GO enrichment (**Supplementary Figure 5**), to evaluate enrichment for DEGs in terms of molecular function, cellular component, and biological process. In terms of biological process, this included metabolic and cellular processes. Cellular components mainly included cells, cell parts, and organelles. Molecular functions mainly included binding and catalytic activities. The results indicate that cells and cell parts were most enriched for DEGs in quinoa leaves, indicating that cellular components played important roles in responding to drought stress. The 50 GO terms with the lowest q values in the enrichment analysis were selected, and the enrichment entries were plotted in a bar chart. The genes showing greater enrichment were related to several biological factors, such as fatty acid metabolic processes, ribosome biogenesis, apoplasts, ribosomal subunits, lyase activity, rRNA binding, and photosynthesis (**Supplementary Figure 6**).

### Analysis of TFs under drought and rewatering conditions

TFs play important roles in plant responses to drought stress and rewatering by regulating the expression levels of target genes. We analyzed TFs associated with the DEGs identified in quinoa leaves. In four comparison groups (**Supplementary Table 13**), 598, 116, 629, and 0 TFs were detected, indicating the key roles of TFs during drought and rewatering treatment. The 1,343 TFs were divided into 55 families. The main TFs in this study included the AP2 (130), MYB (126), bHLH (80), WRKY (79), NAC (64), and bZIP (41) families.

### QRT-PCR validation results

QRT-PCR was used to validate the randomly selected genes. With primers designed by Beacon Designer 7.9 (**Supplementary Table 14**), 17 differentially expressed genes were analyzed using the 2^-ΔΔCт^ method. Compared with log_2_FC, the results show that the gene-*LOC110732446*(Beta-amylase), gene-*LOC110694254*(Beta-amylase), gene*-LOC110688573*(Beta-glucosidase), gene-*LOC110730263*(Beta-amylase), gene-*LOC110684791*(Glucose-1-phosphate adenylyltransferase), gene-*LOC110686667*(Trehalose 6-phosphate synthase), gene-*LOC110710941*(Beta-amylase), gene-*LOC110739236*(Beta-glucosidase), gene-*LOC110693889*(Maltase-glucoamylase), gene-*LOC110696059*(Beta-amylase) had the same up-regulated or down-regulated trend (**Supplementary Table 15**). Ten genes were randomly selected to calculate the relative expression levels of differentially expressed genes by 2^-ΔΔCт.^ This data was compared with log_2_FC values from transcriptome sequencing, and these 10 genes had the same up- or down-regulation trend (**Supplementary Figure 7**). The results showed that the transcriptome sequencing was reliable.

### Analysis of quinoa drought-tolerance mechanisms using combined transcriptomics and metabolomics

**Figure 7:**
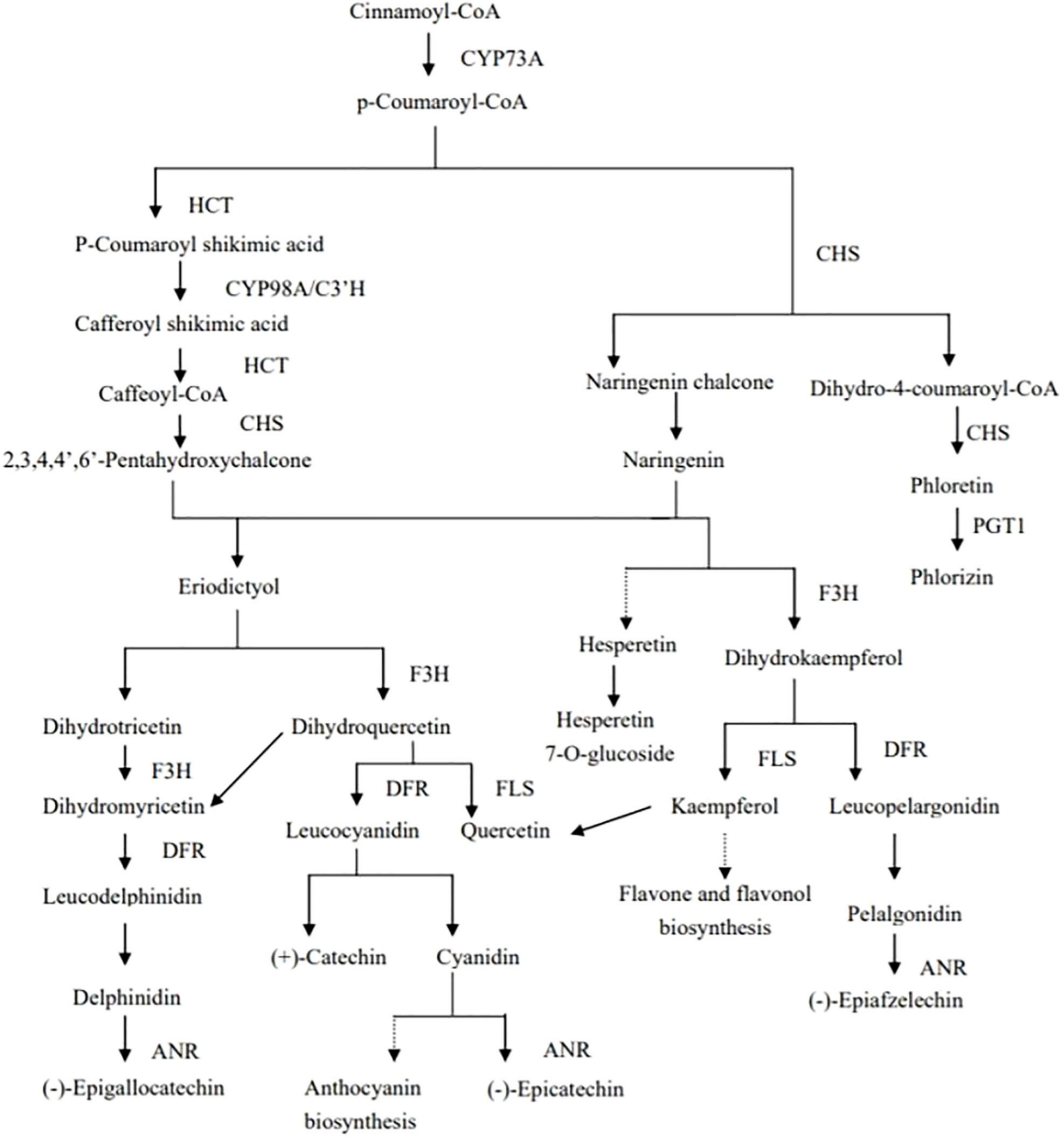
Mechanisms of flavonoid biosynthesis in quinoa.CYP73A is trans-cinnamate 4-monooxygenase, HCT is shikimate O-hydroxycinnamoyl transferase, CYP98A/C3’H is 5-O-(4-coumaroyl)-D-quinate 3’-monooxygenase, CHS is chalcone synthase, PGT1 is phlorizin synthase,F3H is naringenin 3-dioxygenase, DFR is bifunctional dihydroflavonol 4-reductase/flavanone 4-reductase, ANR is anthocyanidin reductase, FLS is flavonol synthase.

Plants can produce a large number of specialized metabolites under drought stress; most of which are antioxidant substances. Among them, flavonoids can improve the drought tolerance of plants under drought stress (Nakabayashi et al., 2014). The biosynthesis of flavonoids originates from Cinnamoyl CoA, and p-coumaroyl-CoA is synthesized through the action of CYP73A. Then, the types and contents of metabolites formed under the action of different enzymes differ, resulting in differences in concentrations of quercetin, hesperetin 7-o-glucoside, kaempferol, and phlorizin, and finally the drought tolerance of quinoa (**Figure 7**). DEGs and DEMs with PCC values of >0.8 in the flavonoid-synthesis pathway were selected. Correlation analysis was performed on the differential genes and differential metabolites, and results with Pearson’s correlation coefficient |PCC| greater than 0.8 were selected, with a positive PCC being a positive correlation and the opposite being a negative correlation. Network diagram of synthetic pathways in flavonoids(**Supplementary Figure 8**), the *LOC110682233* gene was significantly negatively correlated with the metabolites phlorizin, hespertin-7-o-glucoside, kaempferol, and naringenin in DR vs. DC group. The *LOC110703828* gene was significantly negatively correlated with the metabolites phlorizin, hespertin-7-O-glucoside, and kaempferol. The *LOC110713661* gene was significantly positively correlated with kaempferol, phlorizin, and hespertin-7-O-glucoside, The expression of this gene was approximately 20 in different treatments and controls, and the order from high to low was DR, RW, RC and DC, therefore, this may be the key gene for drought tolerance in quinoa. The LOC110729560 gene was significantly negatively correlated with phlorizin (**Supplementary Table 16**). In the RW vs. RC group, the *LOC110722063* gene was significantly positively correlated with phlorizin and hespertin-7-O-glucoside (**Supplementary Table 17**).Through metabolome analysis, we found that phlorizin, naringenin, hespertin-7-O-glucoside, dihydrokaempferol, kaempferol, and quercetin were down-regulated metabolites in the flavonoid-synthesis pathway (**Supplementary Figure 9**). Through transcriptomics analysis, 44, 7, and 51 differentially expressed genes related to flavonoid biosynthesis were found in the DC vs. DR, RC vs. RW, and RW vs. DR comparison groups, respectively (**Supplementary Table 18**). Among these, the *LOC110724467* gene (chalcone synthase, CHS, EC: 2.3.1.74) was down-regulated during drought but stabilized after rewatering. The *LOC110695126* gene (flavanone 3-hydrogenase, F3H, EC: 1.14.11.9) was up-regulated during drought and stabilized after rewatering. The *LOC110709209* gene (5-O-(4-coumaroyl)-D-quinate 3’-monooxygenase, EC: 1.14.14.96) was stable during drought, but up-regulated after rewatering. The *LOC110736236* gene (anthocyanidin reductase, EC: 1.3.1.77) was stably expressed during drought, but down-regulated after rewatering. Eleven genes, including the *LOC110715013* (Shikimate O-hydroxycinnamoyltransferas, EC: 2.3.1.133), were up-regulated or down-regulated during drought, four genes were up-regulated after rewatering, and three genes, including the *LOC110682224* gene (DFR, EC: 1.1.1.219; 1.1.1.234) were up-regulated or down-regulated during drought, but stabilized after rewatering. Eight genes, including the *LOC110708783* gene (FLS, EC: 1.14.20.6), were up-regulated or down-regulated during drought, but stabilized after rewatering (**Supplementary Figure 9**, **Supplementary Tables 19**, **20**). In the flavonoid biosynthesis pathway, the up-regulated expression of enzymes such as F3’H under drought stress, and the stable expression of enzymes such as CHS after rewatering, affected the synthesis of flavonoids, which helps achieve the goal of improving the drought tolerance of quinoa.

Under drought conditions, down-regulated expression of five genes (EC: 2.4.1.357, phlorizin synthase), including the *LOC110693894* gene, promoted the down-regulation of phlorizin. Up-regulated expression of the *LOC110690711* gene and down-regulated expression of four genes, including the *LOC110702757* gene (EC: 5.5.1.6, chalcone isomerase) contributed to naringenin down-regulation. Up-regulated expression of the *LOC110695126* gene (EC:1.14.11.9, naringenin 3-dioxygenase) contributed to dihydrokaempferol down-regulation. Up-regulated expression of three genes, such as the *LOC110698563* gene, and down-regulated expression of five genes, such as the *LOC110699285* gene (EC: 1.14.20.6, FLS) promoted kaempferol and quercetin down-regulation (**Supplementary Figure 9**, **Supplementary Table 19**). The part of the genes shown in **Table 4** were strongly correlated with metabolites in the flavonoid biosynthesis pathway, and the starch- and sucrose metabolism pathways. The *LOC110729560* gene (EC: 2.4.1.357, phlorizin synthase) was significantly negatively correlated with phlorizin, which was down-regulated during drought, but recovered after rewatering. Phlorizin contents were down-regulated during drought but changed minimally after rewatering. KEGG-based functional enrichment of DEGs (transcriptome data) and DEMs (metabolome data) showed that starch and sucrose metabolism were significantly enriched. The DEGs and metabolites with |PCC| >0.8 in the starch- and sucrose metabolism pathways were selected for further analysis. During drought treatment, 32 genes, including the *LOC110715744* gene, were significantly negatively correlated with D-fructose 6-phosphate, whereas the *LOC110712600* gene was significantly positively correlated with D-fructose 6-phosphate. The *LOC110722127* gene and other 12 genes were significantly negatively correlated with D-glucose. Fifteen genes, including the *LOC110695607* gene, were significantly negatively correlated with D-glucose 6-phosphate. The *LOC110738152* gene exhibited a significant negative correlation with the metabolite trehalose 6-phosphate, whereas the *LOC110715744* gene showed a significant positive correlation with trehalose 6-phosphate. Eighteen genes, including the *LOC110687336* gene, showed a significant negative correlation with the metabolite, glucose 1-phosphate (**Supplementary Table 21**). After rehydration, significant negative correlations were found between the *LOC11068475*2 gene and D-sucrose and D-trehalose. The *LOC110721696* gene was significantly positively correlated with D-glucose 6-phosphate (**Supplementary Table 22**).

**Table 4:** Correlation between differential genes and differential metabolites.

Gene	Compounds	PCC	PCCP
*gene-LOC110682233*	Phloretin-2’-O-glucoside (Phlorizin)	-0.859	0.0003
	Hesperetin-7-O-glucoside	-0.884	0.0001
	Kaempferol (3,5,7,4’-Tetrahydroxyflavone)	-0.881	0.0002
	Naringenin (5,7,4’-Trihydroxyflavanone)	-0.828	0.0009
*gene-LOC110703828*	Phloretin-2’-O-glucoside (Phlorizin)	-0.817	0.0012
	Hesperetin-7-O-glucoside	-0.838	0.0007
	Kaempferol (3,5,7,4’-Tetrahydroxyflavone)	-0.865	0.0003
*gene-LOC110713661*	Kaempferol (3,5,7,4’-Tetrahydroxyflavone)	0.844	0.0006
	Phloretin-2’-O-glucoside (Phlorizin)	0.827	0.0009
	Hesperetin-7-O-glucoside	0.807	0.0015
*gene-LOC110729560*	Phloretin-2’-O-glucoside (Phlorizin)	-0.804	0.0016
*gene-LOC110722063*	Phloretin-2’-O-glucoside (Phlorizin)	0.833	0.0008
	Hesperetin-7-O-glucoside	0.814	0.0013
*gene-LOC110682423*	D-Fructose 6-phosphate	-0.824	0.0010
*gene-LOC110686141*	D-Glucose 6-phosphate	-0.885	0.0001
	D-Glucose	-0.84	0.0006
	Glucose-1-phosphate	-0.892	0.0001
	D-Fructose 6-phosphate	-0.919	0.0000
*gene-LOC110686362*	D-Fructose 6-phosphate	-0.836	0.0007
	Glucose-1-phosphate	-0.826	0.0009
	D-Glucose 6-phosphate	-0.81	0.0014
*gene-LOC110686667*	D-Fructose 6-phosphate	0.827	0.0009
*gene-LOC110687336*	Glucose-1-phosphate	-0.904	0.0001
	D-Fructose 6-phosphate	-0.917	0.0000
	D-Glucose 6-phosphate	-0.895	0.0001
	D-Glucose	-0.816	0.0012
*gene-LOC110688727*	Glucose-1-phosphate	-0.82	0.0011
	D-Glucose	-0.812	0.0013
	D-Glucose 6-phosphate	-0.819	0.0011
	D-Fructose 6-phosphate	-0.88	0.0002
*gene-LOC110690443*	D-Fructose 6-phosphate	-0.833	0.0008
*gene-LOC110692291*	D-Glucose	-0.842	0.0006
	D-Fructose 6-phosphate	-0.854	0.0004
*gene-LOC110693540*	Glucose-1-phosphate	-0.804	0.0016
	D-Fructose 6-phosphate	-0.854	0.0004
*gene-LOC110693889*	D-Fructose 6-phosphate	-0.84	0.0006
*gene-LOC110695607*	D-Glucose 6-phosphate	-0.808	0.0015
	D-Fructose 6-phosphate	-0.862	0.0003
	Glucose-1-phosphate	-0.822	0.0010
	D-Glucose	-0.838	0.0007
*gene-LOC110703195*	D-Fructose 6-phosphate	-0.807	0.0015
*gene-LOC110703632*	D-Fructose 6-phosphate	-0.908	0.0000
	Glucose-1-phosphate	-0.86	0.0003
	D-Glucose 6-phosphate	-0.849	0.0005
	D-Glucose	-0.816	0.0012
Gene	Compounds	PCC	PCCP
*gene-LOC110704748*	D-Fructose 6-phosphate	-0.808	0.0015
*gene-LOC110709538*	D-Fructose 6-phosphate	-0.856	0.0004
	D-Glucose 6-phosphate	-0.823	0.0010
	Glucose-1-phosphate	-0.835	0.0007
*gene-LOC110710484*	D-Fructose 6-phosphate	-0.843	0.0006
*gene-LOC110710504*	D-Fructose 6-phosphate	-0.826	0.0009
	Glucose-1-phosphate	-0.803	0.0017
	D-Glucose	-0.815	0.0012
*gene-LOC110711798*	D-Fructose 6-phosphate	-0.834	0.0007
*gene-LOC110712600*	D-Fructose 6-phosphate	0.833	0.0008
*gene-LOC110714985*	Glucose-1-phosphate	-0.834	0.0007
	D-Fructose 6-phosphate	-0.87	0.0002
	D-Glucose 6-phosphate	-0.835	0.0007
*gene-LOC110715744*	D-Fructose 6-phosphate	-0.845	0.0005
	Glucose-1-phosphate	-0.89	0.0001
	Trehalose 6-phosphate	0.804	0.0016
	D-Glucose 6-phosphate	-0.894	0.0001
*gene-LOC110717307*	D-Glucose 6-phosphate	-0.86	0.0003
	Glucose-1-phosphate	-0.858	0.0004
	D-Fructose 6-phosphate	-0.89	0.0001
*gene-LOC110719674*	D-Fructose 6-phosphate	-0.804	0.0016
*gene-LOC110720321*	D-Glucose 6-phosphate	-0.82	0.0011
	Glucose-1-phosphate	-0.826	0.0009
	D-Fructose 6-phosphate	-0.878	0.0002
*gene-LOC110721974*	D-Glucose	-0.818	0.0012
*gene-LOC110722127*	D-Glucose	-0.83	0.0008
	D-Fructose 6-phosphate	-0.845	0.0005
	Glucose-1-phosphate	-0.806	0.0015
*gene-LOC110723159*	D-Glucose 6-phosphate	-0.827	0.0009
	D-Glucose	-0.813	0.0013
	D-Fructose 6-phosphate	-0.874	0.0002
	Glucose-1-phosphate	-0.825	0.0009
*gene-LOC110723897*	D-Fructose 6-phosphate	-0.836	0.0007
*gene-LOC110725158*	D-Glucose 6-phosphate	-0.86	0.0003
	Glucose-1-phosphate	-0.853	0.0004
	D-Glucose	-0.813	0.0013
	D-Fructose 6-phosphate	-0.906	0.0001
*gene-LOC110726353*	D-Fructose 6-phosphate	-0.83	0.0008
	D-Glucose	-0.819	0.0011
*gene-LOC110727926*	D-Glucose 6-phosphate	-0.837	0.0007
	Glucose-1-phosphate	-0.843	0.0006
	D-Fructose 6-phosphate	-0.84	0.0006
*gene-LOC110728048*	D-Fructose 6-phosphate	-0.812	0.0013
*gene-LOC110733459*	D-Glucose 6-phosphate	-0.946	0.0000
	D-Fructose 6-phosphate	-0.91	0.0000
	Glucose-1-phosphate	-0.941	0.0000
*gene-LOC110736307*	D-Fructose 6-phosphate	-0.804	0.0016
*gene-LOC110738152*	Trehalose 6-phosphate	-0.837	0.0007
*gene-LOC110684752*	D-Sucrose	-0.817	0.0012
	D-Trehalose	-0.837	0.0007
*gene-LOC110721696*	D-Glucose 6-phosphate	0.82	0.0011

Through metabolomics analysis, we determined that trehalose-6P, D-glucose-6P, D-fructose-6P, D-glucose, α-D-glucose-1P, trehalose, and sucrose were involved in the starch- and sucrose-metabolic pathways (**Supplementary Figure 10**). Through transcriptome analysis, 187, 30, and 205 differentially expressed genes were identified that were related to starch and sucrose metabolism among the three comparison groups (**Supplementary Table 23**). Three genes, including the *LOC110686362* gene (hexokinase, EC: 2.7.1.1), were down-regulated during drought, but showed stable expression after rewatering. Eight genes, including *LOC110693889* gene (maltase-glucoamylase, EC: 3.2.1.20), were down-regulated during drought, but stably expressed after rehydrating. Four genes, including the *LOC110690627* gene (glucan 1,3-beta-glucosidase, EC: 3.2.1.58), were down-regulated during drought, but stable after rehydration. Three genes, including *LOC110689796* gene (sucrose synthase, EC: 2.4.1.13), were up-regulated during drought, but down-regulated after rehydration. The *LOC110729741* gene (phosphoglucomutase, EC: 5.4.2.2) was down-regulated during drought but stabilized after rehydration. The *LOC110719410* gene (ADP-sugar diphosphatase, EC: 3.6.1.21) was up-regulated during dry drought but stabilized after rehydration. The *LOC110683757* gene (starch synthase, EC: 2.4.1.21) was up-regulated during drought, but down-regulated after rehydration. The *LOC110738898* gene (granule-bound starch synthase, EC: 2.4.1.242) was up-regulated during drought but stabilized after rehydration. Four genes, including *LOC110703195* gene (4-alpha-glucanotransferase, EC: 2.4.1.25), were down-regulated during drought, but stably expressed after rehydrating (**Supplementary Figures 10** and **Supplementary Tables 24**, **25**). In the starch- and sucrose metabolism pathways, the expression levels of enzyme-related genes such as ADP-sugar diphosphatase were up/down-regulated under drought stress, and the expression levels of enzyme-related genes such as hexokinase were restored after rehydration, which enhanced the drought tolerance of quinoa. The *LOC110686362* gene and two other genes were down-regulated (EC: 2.7.1.1, hexokinase), which promoted D-fructose-6P and D-glucose-6P down-regulation. The down-regulated expression of eight genes (EC: 3.2.1.20, maltase-glucoamylase), including the *LOC110693889* gene, promoted the down-regulation of D-glucose, and four genes (EC: 3.2.1.58, glucan 1,3-beta-glucosidase) including the *LOC110690627* gene, promoted the down-regulation of D-glucose. The down-regulation of three genes (EC: 2.4.1.13, sucrose synthase) including the *LOC110689796* gene promoted the down-regulation of sucrose. The down-regulation of the *LOC110729741* gene (EC: 5.4.2.2, phosphoglucomutase) was associated with that of D-glucose-6P. Up-regulation of the *LOC110719410* gene (EC: 3.6.1.21, ADP sugar diphosphatase) promoted the down regulation of α-D-glucose-1P. Down-regulation of the *LOC110703195* gene and three other genes (EC: 2.4.1.25, 4-alpha-glucanotransferase) promoted the down-regulation of D-glucose. Up-regulation of the *LOC110737398* gene (EC: 3.1.3.24, sucrose-6-phosphatase) promoted the up-regulation of sucrose (**Supplementary Figure 10**, **Supplementary Table 24**).Which the *LOC110703632* gene (EC: 2.7.1.4, fructokinase) was significantly negatively correlated with the metabolism of D-fructose 6-phosphate, which was down-regulated during drought, but tended to be steadily expressed after rehydration, whereas the D-fructose 6-phosphate content was down-regulated during drought, but gradually recovered after rehydration. Gene-*LOC110709538* and gene-*LOC110693540* expressed 0 in DR, but low after rehydration. In this study, 42 differential genes (5 for flavonoid biosynthesis and 37 for starch and sucrose metabolism) and 11 differential metabolites (4 for flavonoid biosynthesis and 7 for starch and sucrose metabolism) with differential metabolites were identified as key factors of drought tolerance in quinoa leaves (**Tables 4** –**6**). Gene-*LOC110738152* was not expressed in DC, and was very low or not expressed in RC and RW. This gene was highly expressed in DR. Therefore, gene-*LOC110738152* may be the key gene to improve the drought tolerance of quinoa.

**Table 5:** The log_2_FC of differentially expressed genes.

Gene	EC	Enzyme	Log_2_FC	log_2_FC	log_2_FC RW_vs_DR
			DC_vs _DR	RC_VS_ RW	
*gene-LOC110682233*	1.1.1.219	Dihydroflavonol-4-reductase	-1.5923	-1.1454	-0.0146
*gene-LOC110703828*	2.1.1.104	Caffeoyl-CoA O-methyltransferase	-1.0410	-0.7165	0.1076
*gene-LOC110713661*	2.3.1.133	Shikimate O-hydroxycinnamoyltransferase	1.4368	0.7523	0.2064
*gene-LOC110722063*	2.3.1.133	Shikimate O-hydroxycinnamoyltransferase	0.9084	1.7961	-0.6467
*gene-LOC110729560*	2.4.1.357	Phlorizin synthase	-2.3684	-0.9085	-1.1941
*gene-LOC110686141*	2.4.1.1	Glycogen phosphorylase	-2.1955	-0.8257	-1.234
*gene-LOC110703195*	2.4.1.1	Glycogen phosphorylase	-2.0669	-0.2735	-1.8612
*gene-LOC110711798*	2.4.1.1	Glycogen phosphorylase	-2.4101	-0.6955	-1.823
*gene-LOC110714985*	2.4.1.1	Glycogen phosphorylase	-2.547	-1.1384	-1.3998
*gene-LOC110717307*	2.4.1.1	Glycogen phosphorylase	-1.4565	-0.7021	-0.6242
*gene-LOC110722127*	2.4.1.1	Glycogen phosphorylase	-1.7839	-0.3585	-1.2523
*gene-LOC110686667*	2.4.1.15	Trehalose 6-phosphate synthase	1.3188	0.5304	0.8782
*gene-LOC110686362*	2.7.1.1	Hexokinase	-1.2759	-0.3831	-0.799
*gene-LOC110688727*	2.7.1.1	Hexokinase	-1.848	-0.7427	-1.2488
*gene-LOC110703632*	2.7.1.4	Fructokinase	-1.1064	-0.4192	-0.7366
*gene-LOC110720321*	2.7.1.4	Fructokinase	-1.5628	-0.5718	-1.0074
*gene-LOC110721696*	2.7.1.4	Fructokinase	0.5286	1.3556	-0.7011
*gene-LOC110695607*	3.1.3.12	Trehalose 6-phosphate phosphatase	-1.2305	-0.2609	-0.8932
*gene-LOC110725158*	3.1.3.12	Trehalose 6-phosphate phosphatase	-1.9397	-1.161	-1.0502
*gene-LOC110690443*	3.2.1.1	Alpha-amylase	-1.2586	-0.8134	-0.668
*gene-LOC110728048*	3.2.1.2	Beta-amylase	-1.2861	-0.3646	-1.0604
*gene-LOC110693889*	3.2.1.20	Maltase-glucoamylase	-2.0635	-0.7661	-1.3983
*gene-LOC110687336*	3.2.1.21	Beta-glucosidase	-1.9779	-0.7351	-0.9874
*gene-LOC110693540*	3.2.1.21	Beta-glucosidase	-6.080	-2.6781	/
*gene-LOC110738152*	3.2.1.21	Beta-glucosidase	7.413	0.1203	3
*gene-LOC110684752*	3.2.1.26	Beta-fructofuranosidase	-0.7047	-1.5606	0.187
*gene-LOC110710504*	3.2.1.26	Beta-fructofuranosidase	-2.2885	-0.5805	-1.5052
*gene-LOC110723159*	3.2.1.26	Beta-fructofuranosidase	-2.0124	-0.8969	-1.1699
*gene-LOC110682423*	3.2.1.39	Glucan endo-1,3-beta-D-glucosidase	-3.2789	-0.6438	-2.344
*gene-LOC110704748*	3.2.1.39	Glucan endo-1,3-beta-D-glucosidase	-2.3616	-0.3512	-1.8591
*gene-LOC110710484*	3.2.1.39	Glucan endo-1,3-beta-D-glucosidase	-2.2403	-0.5962	-1.6781
*gene-LOC110715744*	3.2.1.39	Glucan endo-1,3-beta-D-glucosidase	-1.0465	-0.7182	-0.123
*gene-LOC110721974*	3.2.1.39	Glucan endo-1,3-beta-D-glucosidase	-2.6315	-0.5894	-2.3957
*gene-LOC110712600*	3.2.1.4	Endoglucanase	2.1741	0.8014	1.6283
Gene	EC	Enzyme	Log_2_FC	log_2_FC	log_2_FC RW_vs_DR
			DC_vs _DR	RC_VS_ RW	
*gene-LOC110719674*	3.2.1.4	Endoglucanase	-1.2416	-0.1616	-0.9423
*gene-LOC110723897*	3.2.1.4	Endoglucanase	-3.1699	-1.1673	-1.8015
*gene-LOC110726353*	3.2.1.4	Endoglucanase	-4.812	-0.848	/
*gene-LOC110727926*	3.2.1.4	Endoglucanase	-1.2437	-0.4567	-0.5519
*gene-LOC110733459*	3.2.1.4	Endoglucanase	-3.0444	-2.663	-0.585
*gene-LOC110736307*	3.2.1.4	Endoglucanase	-3.0686	-0.5679	-2.8164
*gene-LOC110709538*	3.2.1.58	Glucan 1,3-beta-glucosidase	-6.174	-1.2345	/
*gene-LOC110692291*	3.2.1.68	Isoamylase	-2.7769	-0.8795	-2.0478

**Table 6:** The log_2_FC of differential metabolites.

Compounds	Log_2_FC DC_vs _DR	log_2_FC RC_VS_ RW	log_2_FC RW_vs_DR
Phlorizin	-1.7576	-2.0336	0.1234
Kaempferol	-3.1101	-2.0164	-1.4189
Hesperidin-7-O-glucoside	-1.5406	-1.7914	-0.1764
Naringenin	-12.4423	-3.1737	-10.0105
D-Glucose 6-phosphate	-1.7741	-1.5406	-0.0440
D-Fructose 6-phosphate	-1.6879	-1.4563	0.2797
D-Glucose	-1.0217	-0.7181	0.2220
Glucose 1-phosphate	-1.8962	-1.4918	-0.1369
Trehalose 6-phosphate	2.0636	0.2035	1.4972
D-Sucrose	-0.9879	-2.9574	2.3223
D-Trehalose	-1.0142	-2.5478	1.8593

## Discussion

Water scarcity has devastating effects on the yield and quality of major crops, and water deficits caused by drought can lead to severe growth retardation and yield loss (Zhang et al, 2014). When plants are exposed to a water deficit, they undergo highly complex morphological, physiological, biochemical, and molecular changes (Bhargava and Sawant, 2013). Drought stress can reduce plant heights, ear lengths, chlorophyll contents, and root and stem biomass, thereby reducing grain yields (Abbas et al., 2018). In the present study, similar conclusions to previous studies were reached, whereby several parameters of the morphology of the drought tolerant genotype Dianli 129 showed increase or a small decrease under drought conditions. The ability to maintain key biological functions during drought and recover quickly after rewatering are important determinants of the maximum lifetime productivity and high drought tolerance (Abid et al., 2018). Dianli 129 showed better recovery ability after rewatering, and its drought tolerance mechanism may occur by reducing the above-ground part biomass and leaf area while maintaining a larger root-to-crown ratio to better maintain normal growth. In contrast, the drought-sensitive genotype Dianli 114 demonstrated a decline in total root length and total root volume under drought and did not recover well after rehydration, likely because drought stress inhibited root growth. Photosynthesis is enhanced under drought stress, which confers a high potential to withstand drought stress (Abid et al., 2016). The physiological changes of tea plants under drought and rehydration conditions have been studied (Liu et al., 2015). With the development of drought stress, the MDA, SS, and Pro contents, as well as the SOD and CAT activities increased significantly, however, decreased rapidly after rehydration. The ABA and SA levels peaked at an early stage of drought stress and then declined rapidly (Liu et al., 2015). Pro, total SS, ascorbic acid, and ABA levels increased in the drought-tolerant varieties, whereas hydrogen peroxide, superoxide anions, lipid peroxidation, and electrolyte leakage increased rapidly in the drought-sensitive varieties, indicating that the tolerant varieties showed higher antioxidant capacities and stronger protective mechanisms (Das et al., 2015). Proteins rich in glutathione, taurine, hypotaurine, methionine, cysteine, and other amino acids involved in sulfur-dependent metabolic pathways were significantly altered under drought stress (Wang et al., 2017). In this study, the drought tolerant genotype Dianli 129 maintained a high total antioxidant capacity under drought. Soluble protein content increased sharply under drought conditions, which may be closely related to the assay method, varietal differences, stress intensity and time. Relative conductivity increased less, chlorophyll content also decreased less and recovered better or showed supercompensation after rehydration. Under drought stress and rehydration, the drought tolerant genotype Dianli 129 showed higher enzyme activity and higher osmoregulatory substances, and therefore exhibited a greater ability to resist drought stress as well as recover after rehydration. This genotype could be used as an important drought tolerant quinoa germplasm resource.

In recent years there have been many histological studies demonstrating that plant resistance is associated with flavonoids. Transcriptome and metabolite analysis of grapes under drought stress showed that water scarcity regulated the expression of structural genes related to phenylpropane, flavonoids, carotenoids, and terpenoids, and these metabolic pathways underwent transcriptional regulation in grapes under water stress (Savoi et al., 2016). Comparative transcriptome analysis of *Ammopiptanthus mongolicus* under drought and cold stress revealed that flavonoid biosynthesis genes were enriched in DEG up-regulated by both stresses (Wu et al., 2014). The effects of fulvic acid on genes and metabolites of tea plants during different drought stress stages were studied by transcriptomics and metabolomics. The results showed that fulvic acid could enhance ascorbic acid metabolism, improve glutathione metabolism, and promote the biosynthesis of flavonoids (e.g., C4H, CHS, F3’5’h, F3H, kaempferol, and quercetin). Thus, fulvic acid could significantly improve the antioxidant-defense abilities of tea plants under drought stress, to enhance the drought tolerance of tea plants (Sun et al., 2020). Sugars (sucrose and trehalose) influence the regulation of cell osmotic pressure during the stress response of plants during drought (Shinozaki and Yamaguchi-Shinozaki, 2007). Similar to previous studies in the present study, DEGs were analyzed using the KEGG database, and the haircut showed a significant enrichment of KEGG metabolite pathways including flavonoid biosynthesis, suggesting that possibly quinoa drought tolerance is also associated with flavonoids. The gene gene-LOC110713661 was significantly positively correlated with flavonoids (Kaempferol, Phlorizin, Hesperetin-7-O-glucoside), which means that this gene promotes the synthesis of flavonoids to tolerate drought. However, gene-LOC110738152 was highly expressed in the drought treatment, but not in the drought control, and had very low or no expression in the rewatering treatment and the rewatering control. We therefore infer that gene-LOC110713661 and gene-LOC110738152 may be key genes for enhancing drought tolerance in quinoa. The tolerance to drought stress may be due to the elevated expression levels of these two genes, and the drought tolerance genes may still be lowly expressed or slowly not expressed after later rewatering due to environmental changes. This study involved six metabolites (phlorizin, naringenin, hespertin-7-O-glucoside, dihydrokaempferol, kaempferol, and quercetin), as well as CHS, naringenin 3-dioxygenase, anthocyanidin reductase, and FLS, among other enzymes. Previous data showed that DREB, ERF, NAC, and WRKY were jointly regulated by drought and cold stress (Wu et al., 2014). The main TFs examined in this study include AP2, MYB, BHLH, WRKY, NAC, and bZIP. The gene expression levels may be related to drought tolerance in quinoa. Trehalose-6p, D-glucose-6P, D-fructose-6P, D-glucose, α-D-glucose-1P, trehalose, and sucrose, as well as related genes such as hexokinase, fructokinase, malpase-glucoamylase, and glucan 1,3-beta-glucosidase, may be correlated with drought tolerance in quinoa. In this study, 42 DEGs and 11 DEMs were identified as key factors of drought tolerance in quinoa leaves. We also discovered 3,259 genes potentially related to drought tolerance in quinoa (**Supplementary Table 26**).

## Conclusion

To study the mechanisms mediating responses to drought stress at the seedling stage in quinoa, we adopted a drought stress–rehydration method and conducted transcriptomics and metabolomics analyses on the drought-tolerant Dianli 129 quinoa genotype. The results showed that the gene-*LOC110713661* and gene-*LOC110738152* may be key genes for drought tolerance in quinoa, and our findings provide a theoretical basis for breeding drought-tolerant quinoa genotypes. In this study, we investigated the biosynthetic pathways of flavonoids in quinoa and the active roles of the starch- and sucrose metabolism pathways in quinoa under drought stress. The results of this study confirm that a strategy to protect quinoa from drought stress is to regulate the antioxidant systems and the accumulation of metabolites. The Dianli 129 quinoa genotype showed a high ability to resist drought stress and to recover after rehydration.

## Data availability statement

The data presented in the study are deposited in the NCBI repository, accession number PRJNA857812.

## Author contributions

XH: Writing - Original Draft, Methodology. LL: Conceptualization, Writing- Review & Editing. YL: Formal analysis, Methodology. ZK: Data Curation, Visualization. YL: Data Curation, Investigation. QW, JL: Methodology, Visualization. PZ,YG: Formal analysis, Investigation. PQ: Supervision, Project administration, Funding acquisition. All authors contributed to the article and approved the submitted version.

## Funding

This research was funded by Yunnan Academician Workstation (2019IC006), Central Government for Guiding Local Science and Technology Development (2020, Quinoa), and the Kunming Science and Technology Innovation Center (2019-1-N-25318000002317).

## Acknowledgments

We thank the staff of Wuhan Metware Biotechnology Co., Ltd.(Wuhan, China), for their support during the metabolite data analysis. We would like to thank Editage (www.editage.cn) for English language editing.

## Conflict of interest

The authors declare that the research was conducted in the absence of any commercial or financial relationships that could be construed as a potential conflict of interest.

## Publisher’s note

All claims expressed in this article are solely those of the authors and do not necessarily represent those of their affiliated organizations, or those of the publisher, the editors and the reviewers. Any product that may be evaluated in this article, or claim that may be made by its manufacturer, is not guaranteed or endorsed by the publisher.

## Abbreviations

ABA, abscisic acid; CHS, chalcone synthase; DC, drought control; DEG, differentially expressed gene; DEM, differentially expressed metabolite; DFR, dihydroflavonol 4-reductase; DR, drought treatment; EC, Enzyme Commission; F3H, flavanone 3-hydrogenase; FC, fold-change; FPKM, fragments per kilobase of transcript, per million mapped reads; GO, Gene Ontology; JA, jasmonates; KEGG, Kyoto Encyclopedia of Genes and Genomes; KOG, Eukaryotic Orthologous Group; MDA, malondialdehyde; PCA, principal component analysis; PCC, Pearson’s correlation coefficient; POD, peroxidase; Pro, proline; RC, rewatered control; RW, rewatering treatment; SA, salicylic acid; SOD, superoxide dismutase; SS, soluble sugar; TF, transcription factor TIC, total ion current.
