# Supplementary material for: Integrating transcriptomics and metabolomics to analyze quinoa (Chenopodium quinoa Willd.) responses to drought stress and rewatering
Source: Front Plant Sci. 2022 Oct 26;13:988861. doi: 10.3389/fpls.2022.988861 (PMC9645111; doi:10.3389/fpls.2022.988861)
Supplement: Supplementary file 1 [file DataSheet_1.zip › Supplementary materials/Supplementary Figure 1.docx]

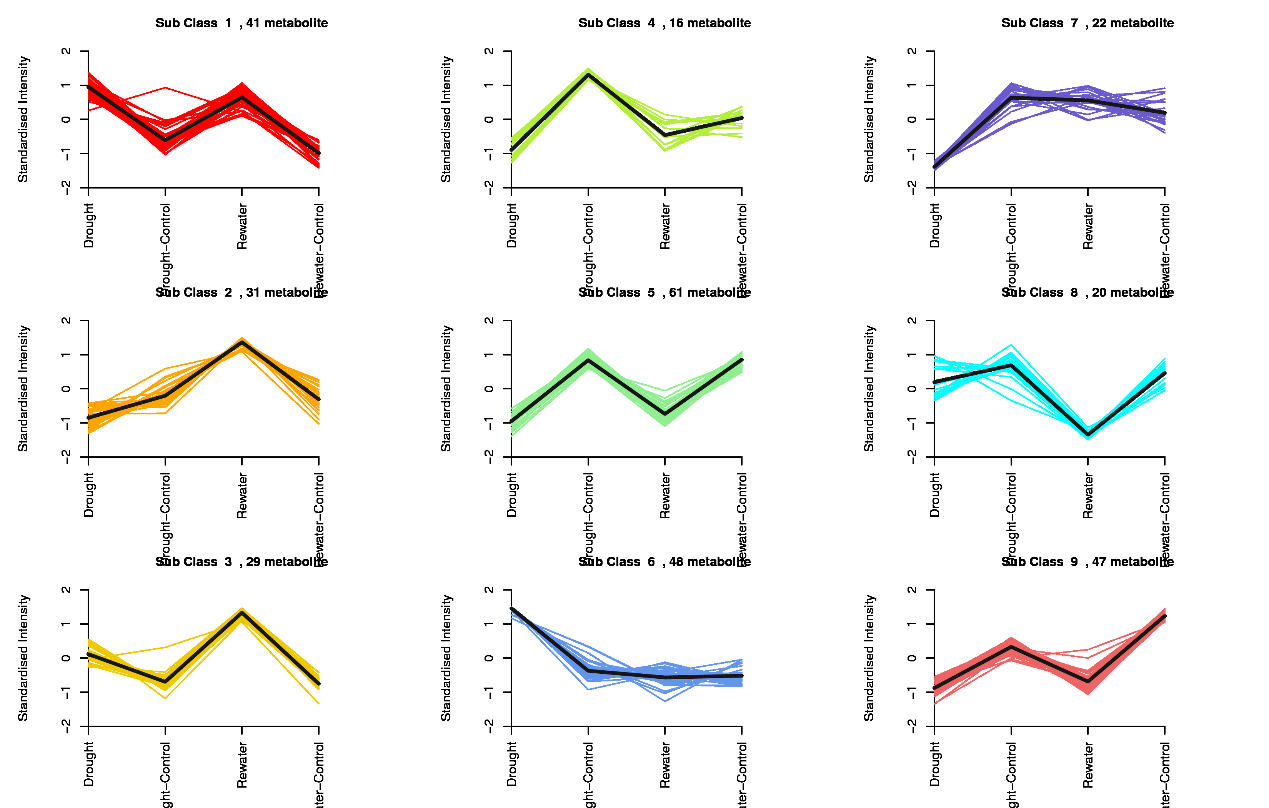


Figure S1. Kmeans diagram of differential metabolites

Note: the abscissa represents the name of the sample, the ordinate represents the relative content of the standardized metabolites, “sub class” represents the number of metabolites with the same change trend, and “* metabolite” represents the number of metabolites in this category.
