# Supplementary material for: Integrating transcriptomics and metabolomics to analyze quinoa (Chenopodium quinoa Willd.) responses to drought stress and rewatering
Source: Front Plant Sci. 2022 Oct 26;13:988861. doi: 10.3389/fpls.2022.988861 (PMC9645111; doi:10.3389/fpls.2022.988861)
Supplement: Supplementary file 1 [file DataSheet_1.zip › Supplementary materials/Supplementary Figure 2.docx]

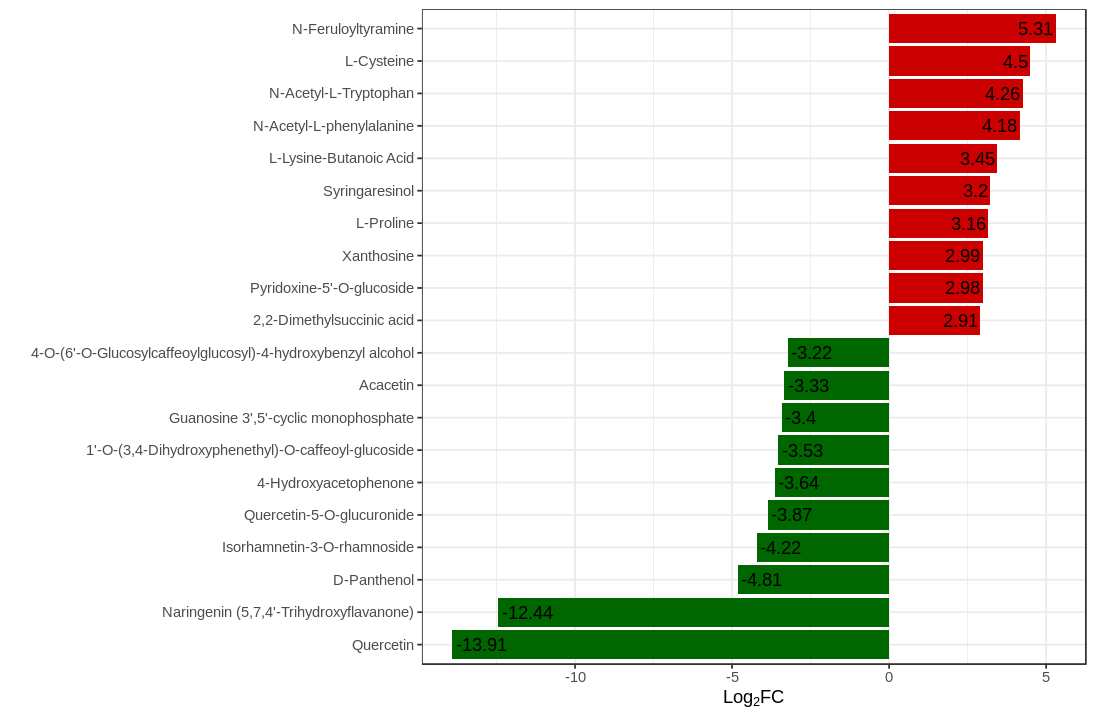


**A**


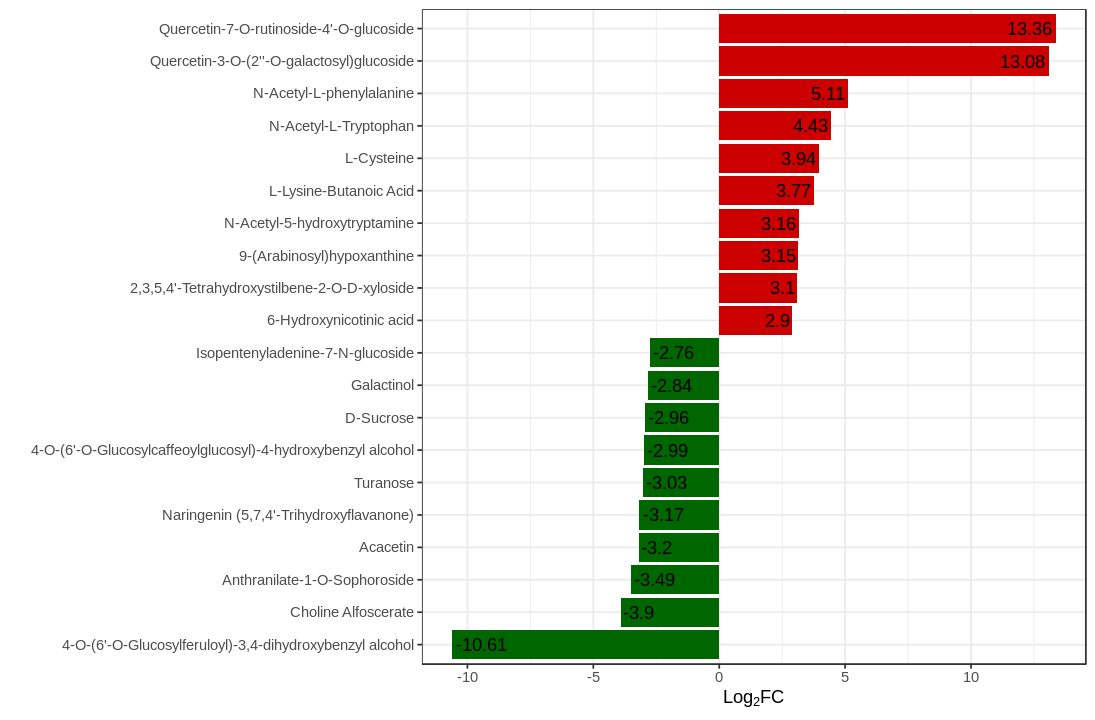


**B**


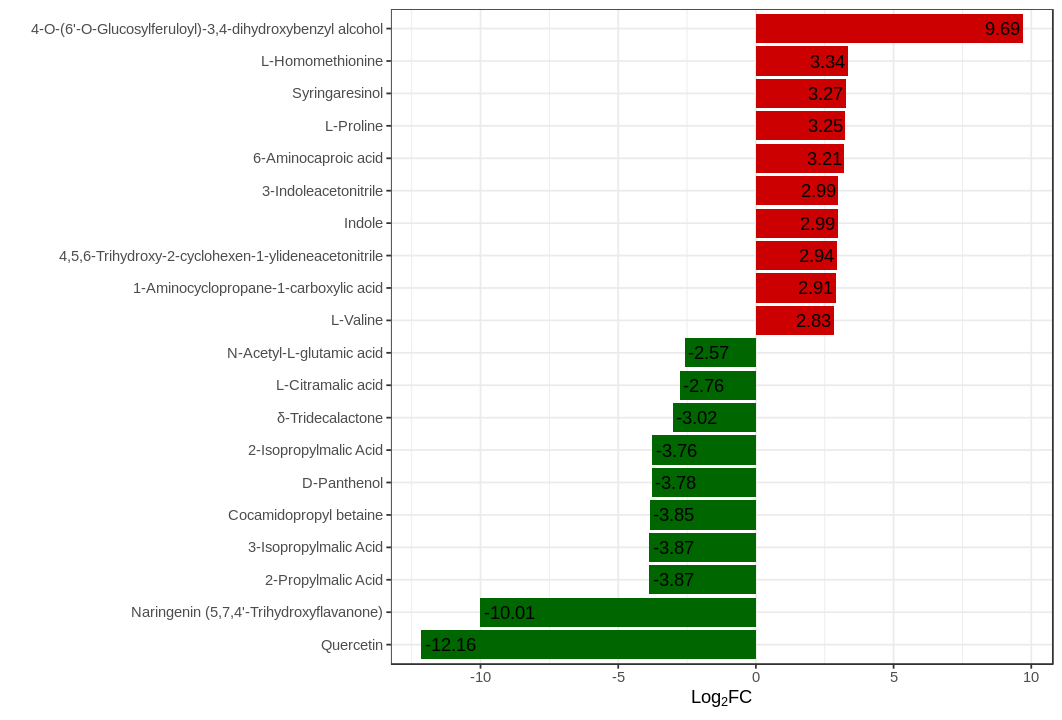


**C**

**D**


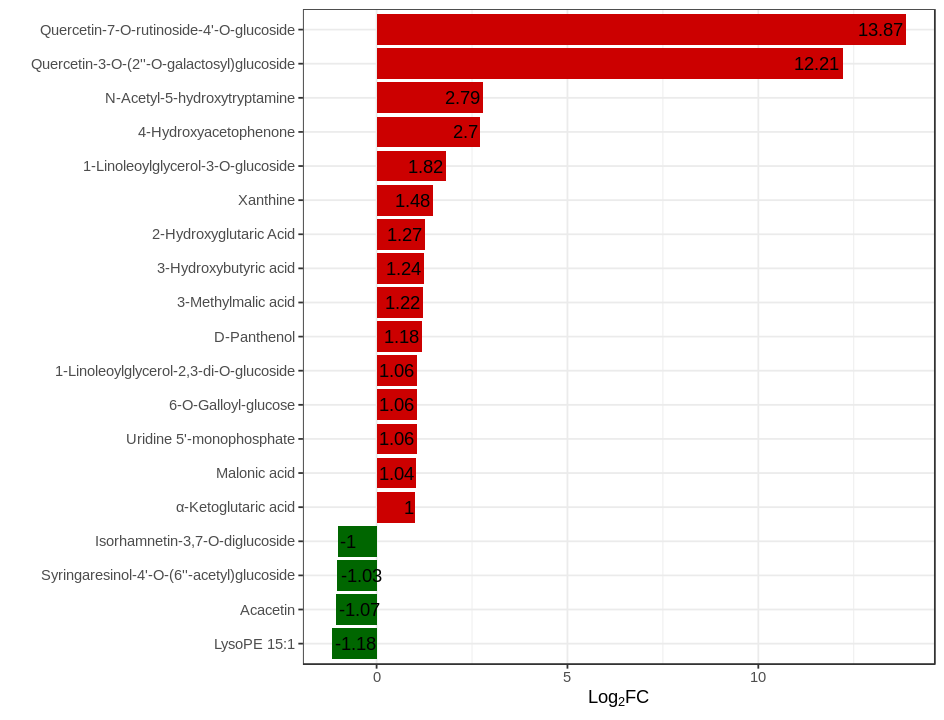


Figure S2. Histogram of multiple of differences

Note: A. Drought-Control_vs_Drought；B. Rewater-Control_vs_Rewater; C.Rewater_vs_Drought; D. Dought-Control_vs_Rewater-Control.The abscissa is the log_2_FC of the different metabolites, that is, the difference multiple of the different metabolites, taking the logarithm value as the base 2, and the ordinate is the difference metabolites. Red represents up regulation of differentially expressed metabolites and green represents down regulation of differentially expressed metabolites
