# Supplementary material for: Integrating transcriptomics and metabolomics to analyze quinoa (Chenopodium quinoa Willd.) responses to drought stress and rewatering
Source: Front Plant Sci. 2022 Oct 26;13:988861. doi: 10.3389/fpls.2022.988861 (PMC9645111; doi:10.3389/fpls.2022.988861)
Supplement: Supplementary file 1 [file DataSheet_1.zip › Supplementary materials/Supplementary Figure 3.docx]

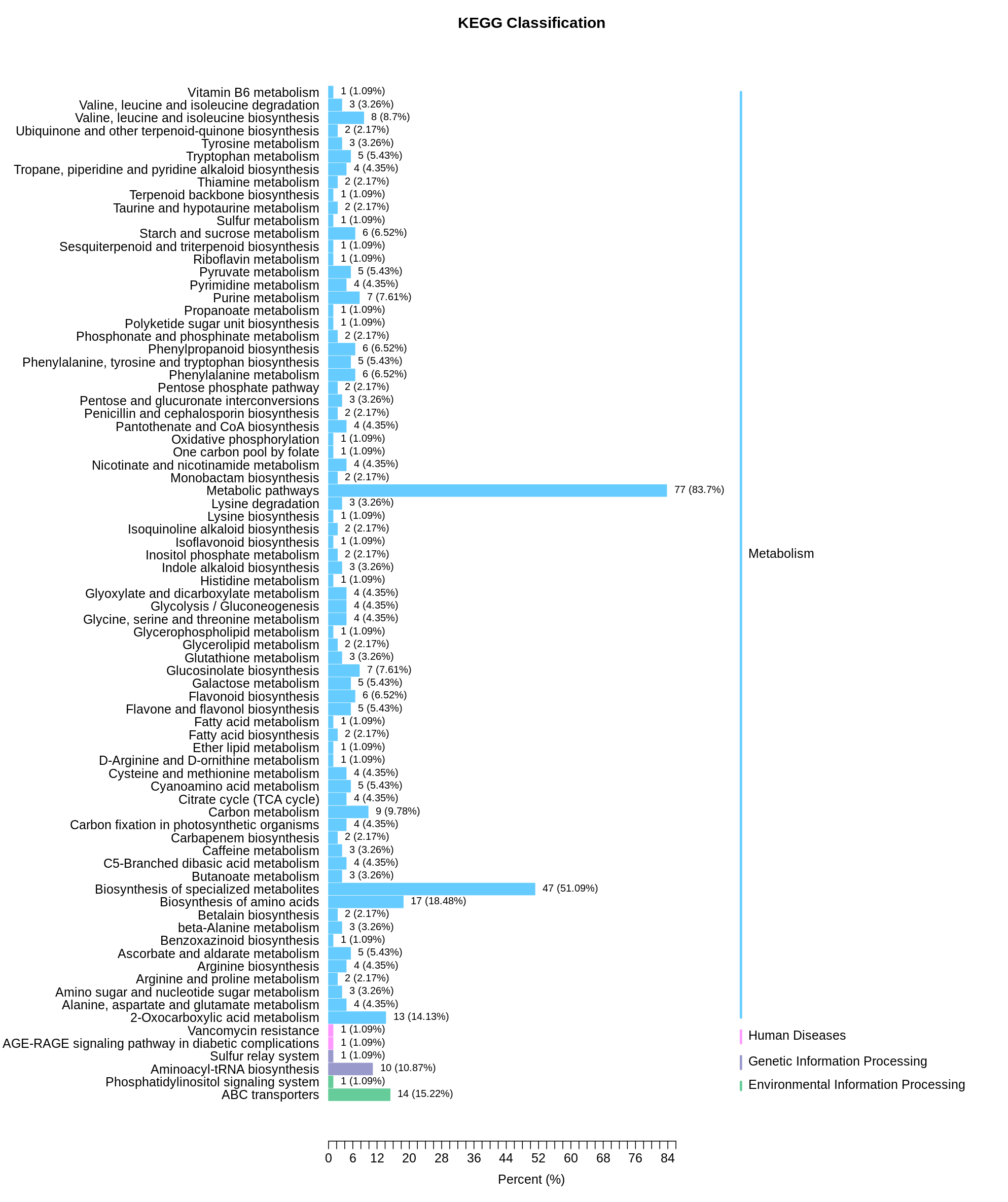


**A**


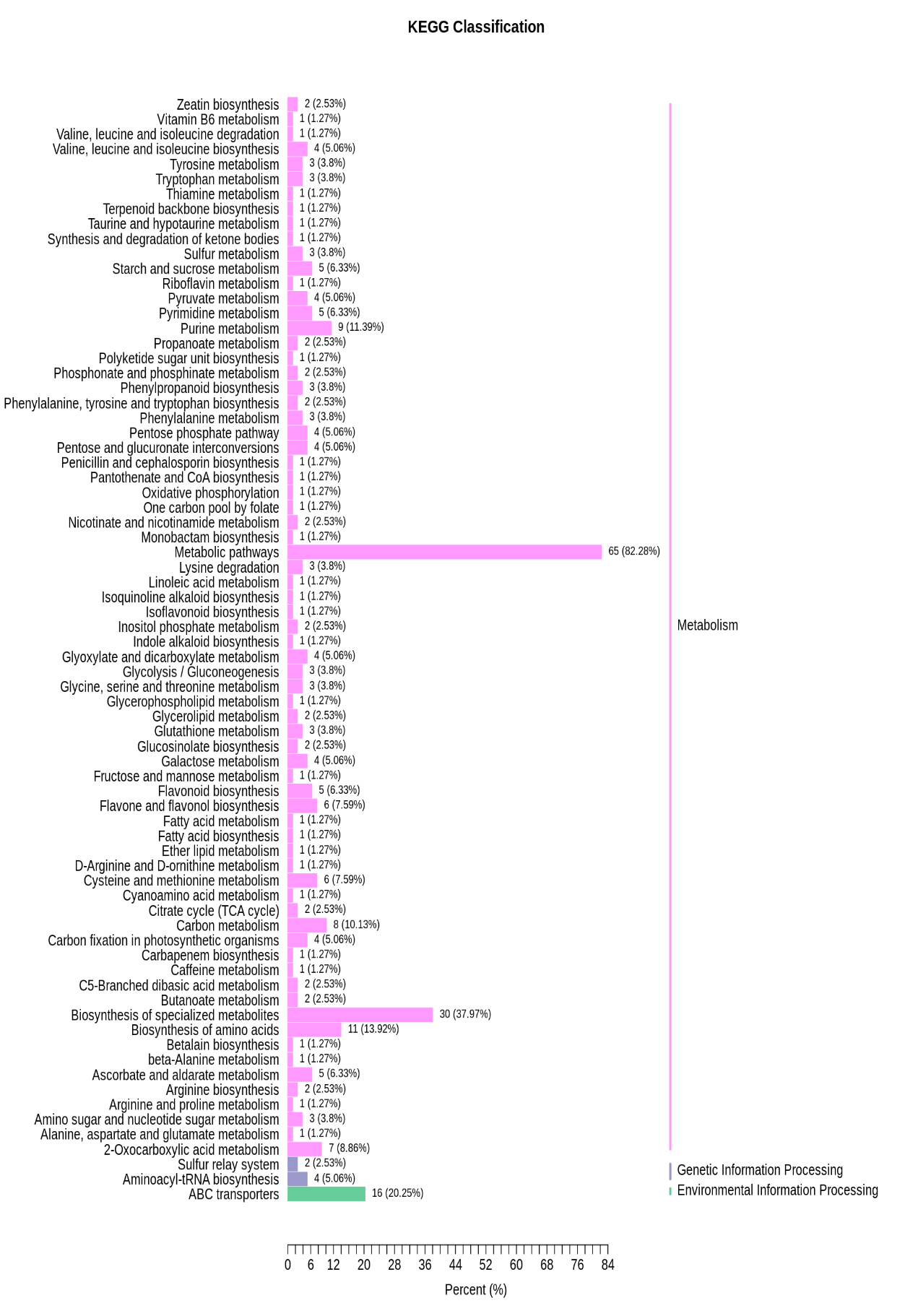


B


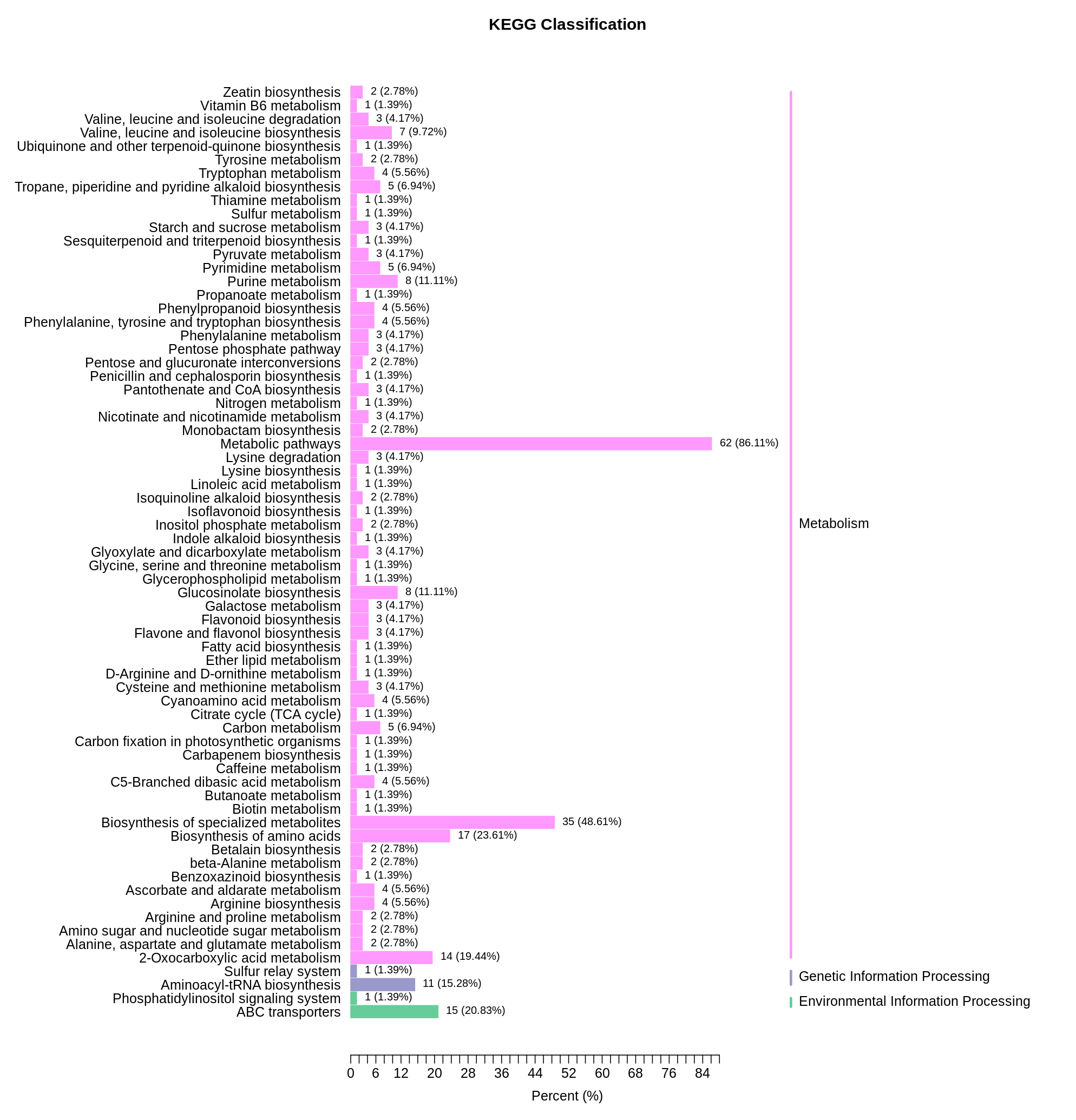


C


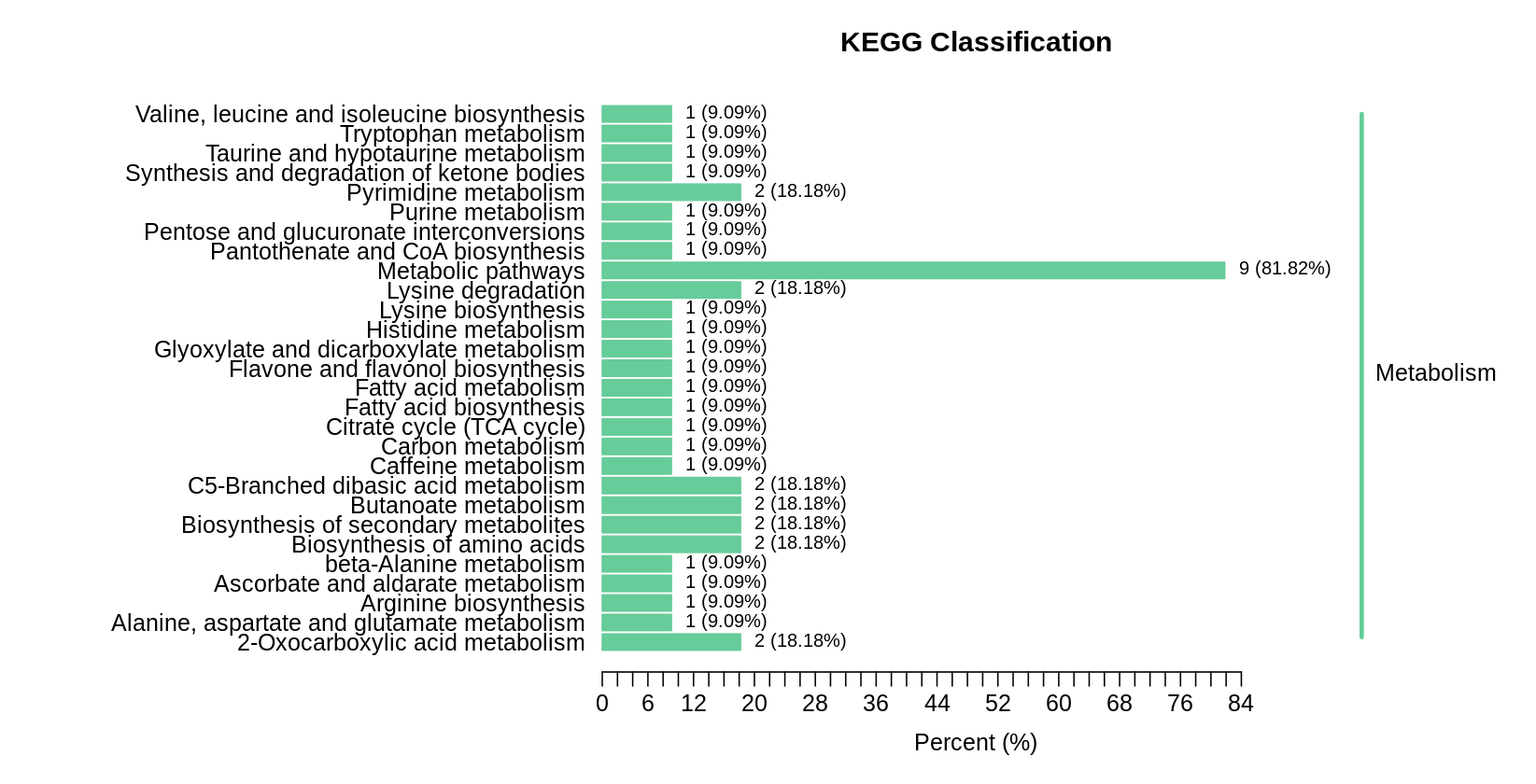


D

Figure S3. KEGG classification map of different metabolites

Note: A. Drought-Control_vs_Drought；B. Rewater-Control_vs_Rewater; C.Rewater_vs_Drough; D. Dought-Control_vs_Rewater-Control. The ordinate is the name of KEGG metabolic pathway, and the abscissa is the number of metabolites annotated to the pathway and its proportion to the total number of metabolites annotated.
