# Supplementary material for: Integrating transcriptomics and metabolomics to analyze quinoa (Chenopodium quinoa Willd.) responses to drought stress and rewatering
Source: Front Plant Sci. 2022 Oct 26;13:988861. doi: 10.3389/fpls.2022.988861 (PMC9645111; doi:10.3389/fpls.2022.988861)
Supplement: Supplementary file 1 [file DataSheet_1.zip › Supplementary materials/Supplementary Figure 4.docx]

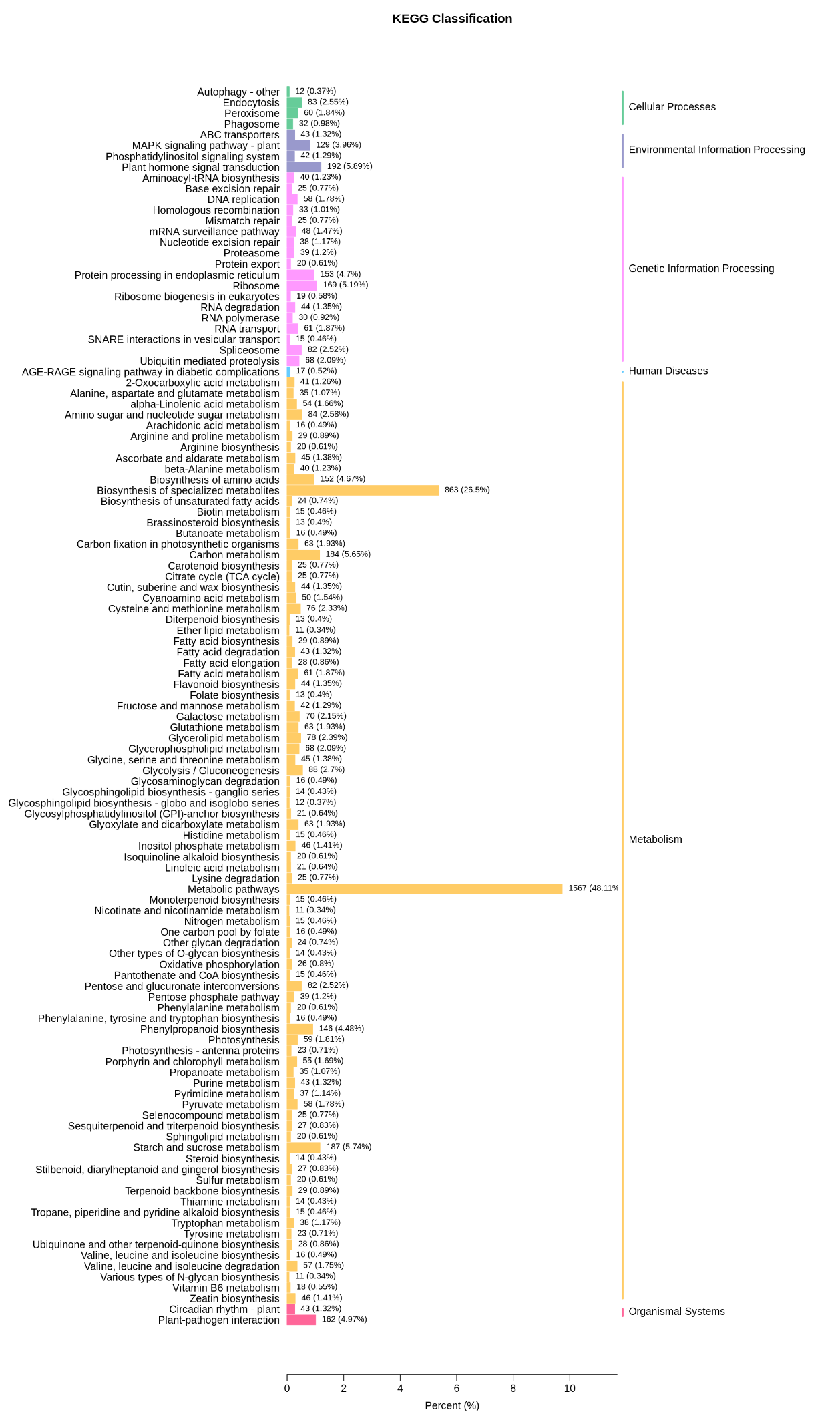


A


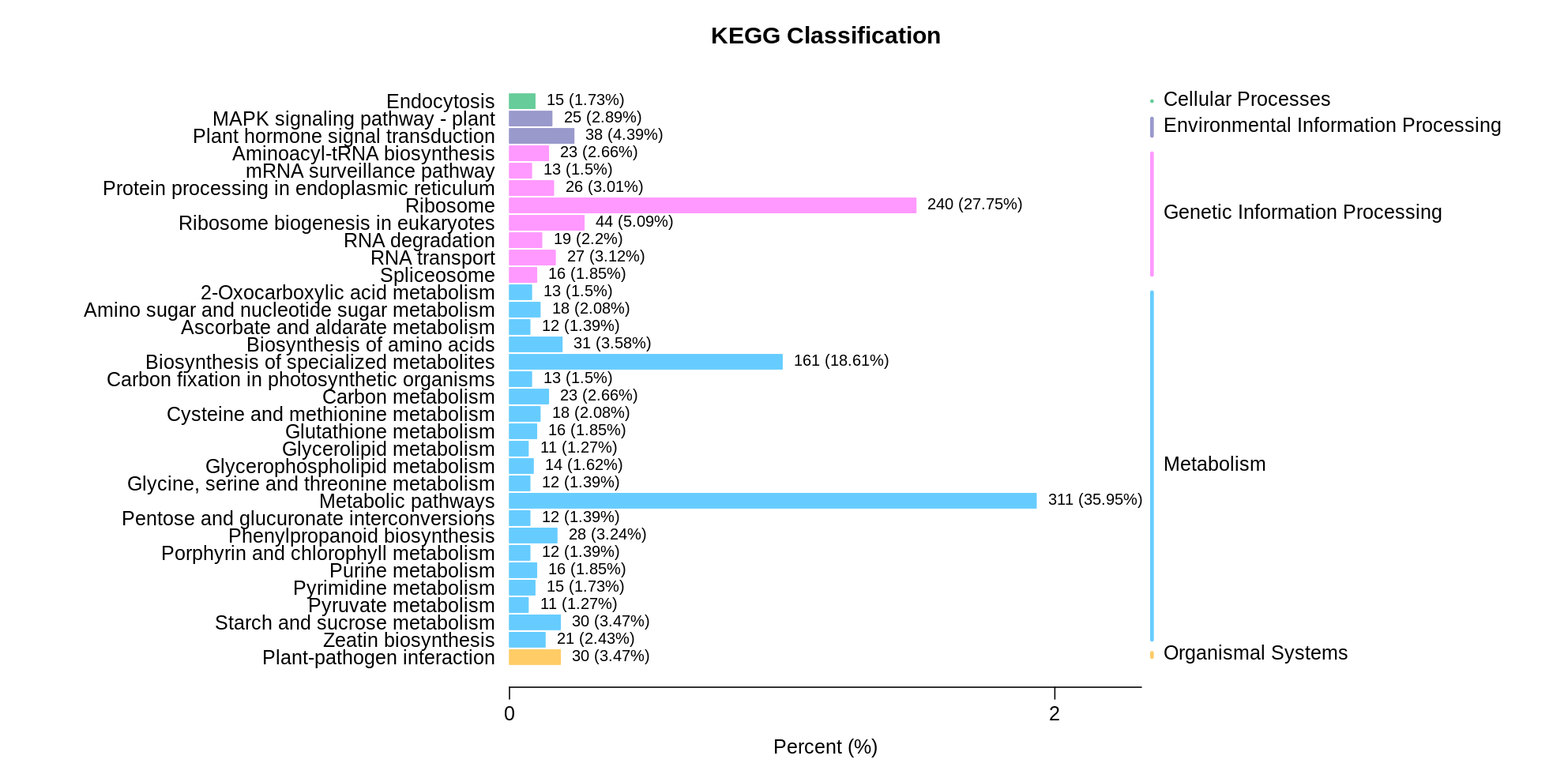


B


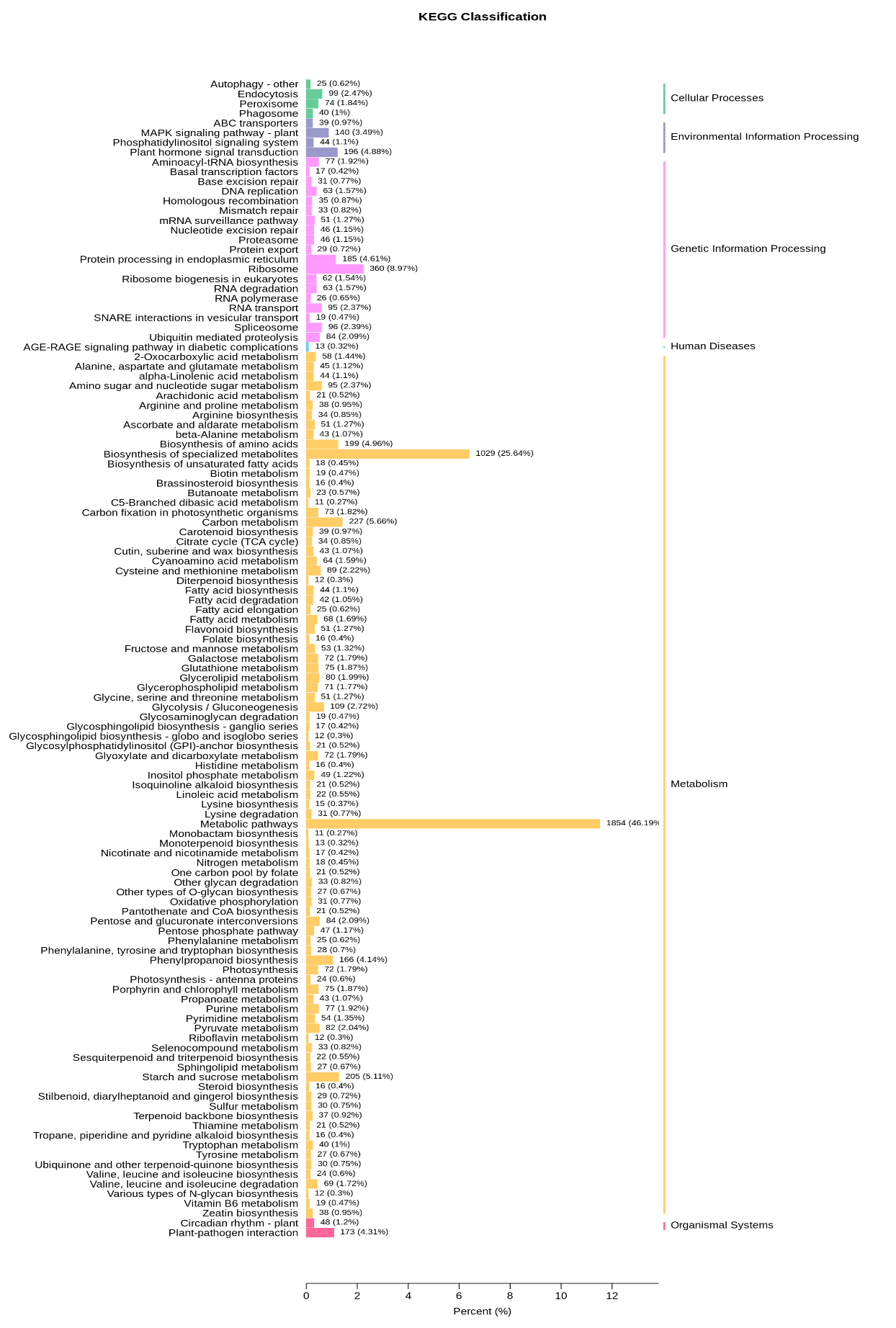


C

Figure S4. KEGG classified column chart

Note: A. Drought-Control_vs_Drought；B. Rewater-Control_vs_Rewater; C.Rewater_vs_Drought. The abscissa represents the proportion of genes annotated to the pathway to the total number of annotated genes, and the ordinate represents the name of KEGG pathway. The label on the right side of the graph represents the classification of KEGG pathway.
