# Supplementary material for: Integrating transcriptomics and metabolomics to analyze quinoa (Chenopodium quinoa Willd.) responses to drought stress and rewatering
Source: Front Plant Sci. 2022 Oct 26;13:988861. doi: 10.3389/fpls.2022.988861 (PMC9645111; doi:10.3389/fpls.2022.988861)
Supplement: Supplementary file 1 [file DataSheet_1.zip › Supplementary materials/Supplementary Figure 5.docx]

| 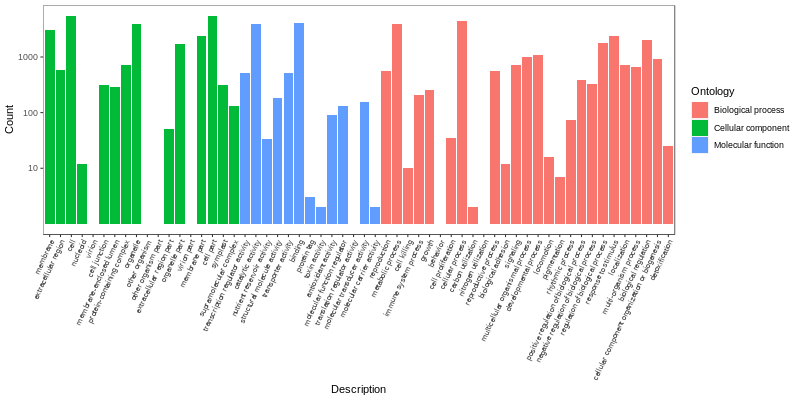  C  A | 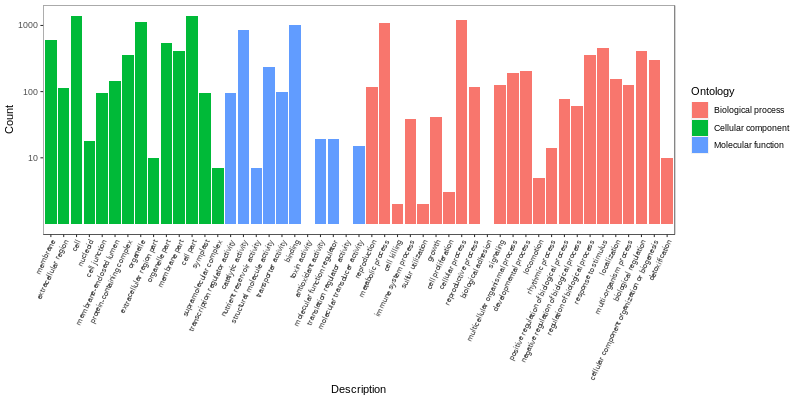  D  B |
| --- | --- |
| 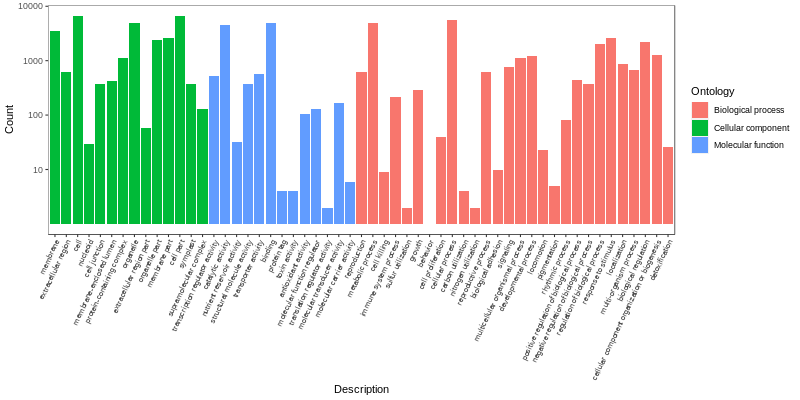 | 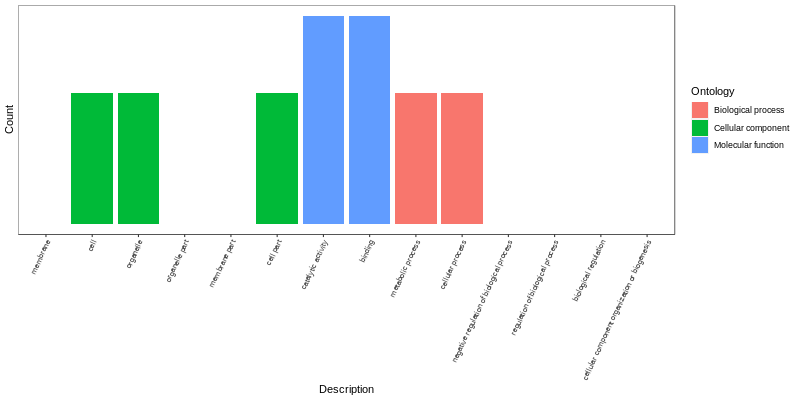 |

Figure S5. Go classification map of differentially expressed genes

Note: A. Drought-Control_vs_Drought；B. Rewater-Control_vs_Rewater; C.Rewater_vs_Drought; D. Dought-Control_vs_Rewater-Control. The abscissa represents the secondary GO items, and the ordinate represents the number of differential genes of GO items.
