# Supplementary material for: Integrating transcriptomics and metabolomics to analyze quinoa (Chenopodium quinoa Willd.) responses to drought stress and rewatering
Source: Front Plant Sci. 2022 Oct 26;13:988861. doi: 10.3389/fpls.2022.988861 (PMC9645111; doi:10.3389/fpls.2022.988861)
Supplement: Supplementary file 1 [file DataSheet_1.zip › Supplementary materials/Supplementary Figure 7.docx]

Figure S7. Relative expression level of differentially expressed genes

gene-LOC110732446, Beta-amylase

gene-LOC110694254, Beta-amylase

gene-LOC110693049, Trehalose 6-phosphate synthase

gene-LOC110686667, Trehalose 6-phosphate synthase

gene-LOC110684791, Glucose-1-phosphate adenylyltransferase

gene-LOC110729560, Phlorizin synthase

gene-LOC110703632, Fructokinase

gene-LOC110710504, Beta-fructofuranosidase

gene-LOC110739236, Beta-glucosidase

gene-LOC110693889, Maltase-glucoamylase
