# Supplementary material for: Integrating transcriptomics and metabolomics to analyze quinoa (Chenopodium quinoa Willd.) responses to drought stress and rewatering
Source: Front Plant Sci. 2022 Oct 26;13:988861. doi: 10.3389/fpls.2022.988861 (PMC9645111; doi:10.3389/fpls.2022.988861)
Supplement: Supplementary file 1 [file DataSheet_1.zip › Supplementary materials/Supplementary Figure 8.docx]

| 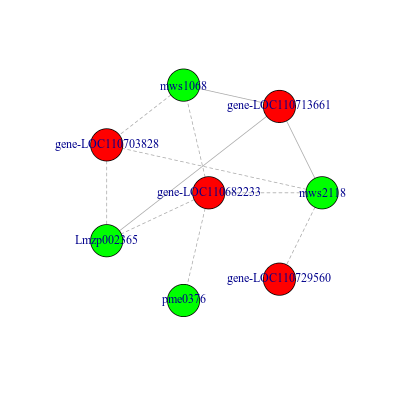  A | 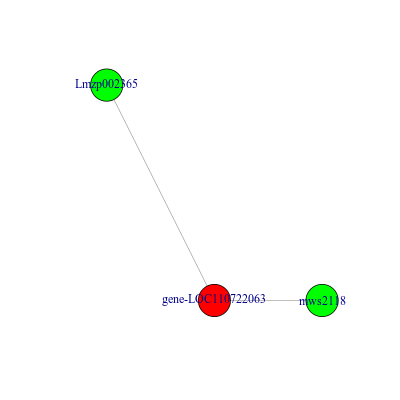  B |  |
| --- | --- | --- |

Figure S8. Correlation network diagram.

A. Drought-Control_vs_Drought；B. Rewater-Control_vs_Rewater.

The correlation between metabolites and genes is expressed by network graph, and the differential genes and differential metabolites with correlation greater than 0.8 in each pathway are selected for mapping. In the figure, metabolites are marked with green, genes are marked with red, solid line represents positive correlation, and dotted line represents negative correlation.
