# Supplementary material for: Integrating transcriptomics and metabolomics to analyze quinoa (Chenopodium quinoa Willd.) responses to drought stress and rewatering
Source: Front Plant Sci. 2022 Oct 26;13:988861. doi: 10.3389/fpls.2022.988861 (PMC9645111; doi:10.3389/fpls.2022.988861)
Supplement: Supplementary file 1 [file DataSheet_1.zip › Supplementary materials/Supplementary Figure 9.docx]

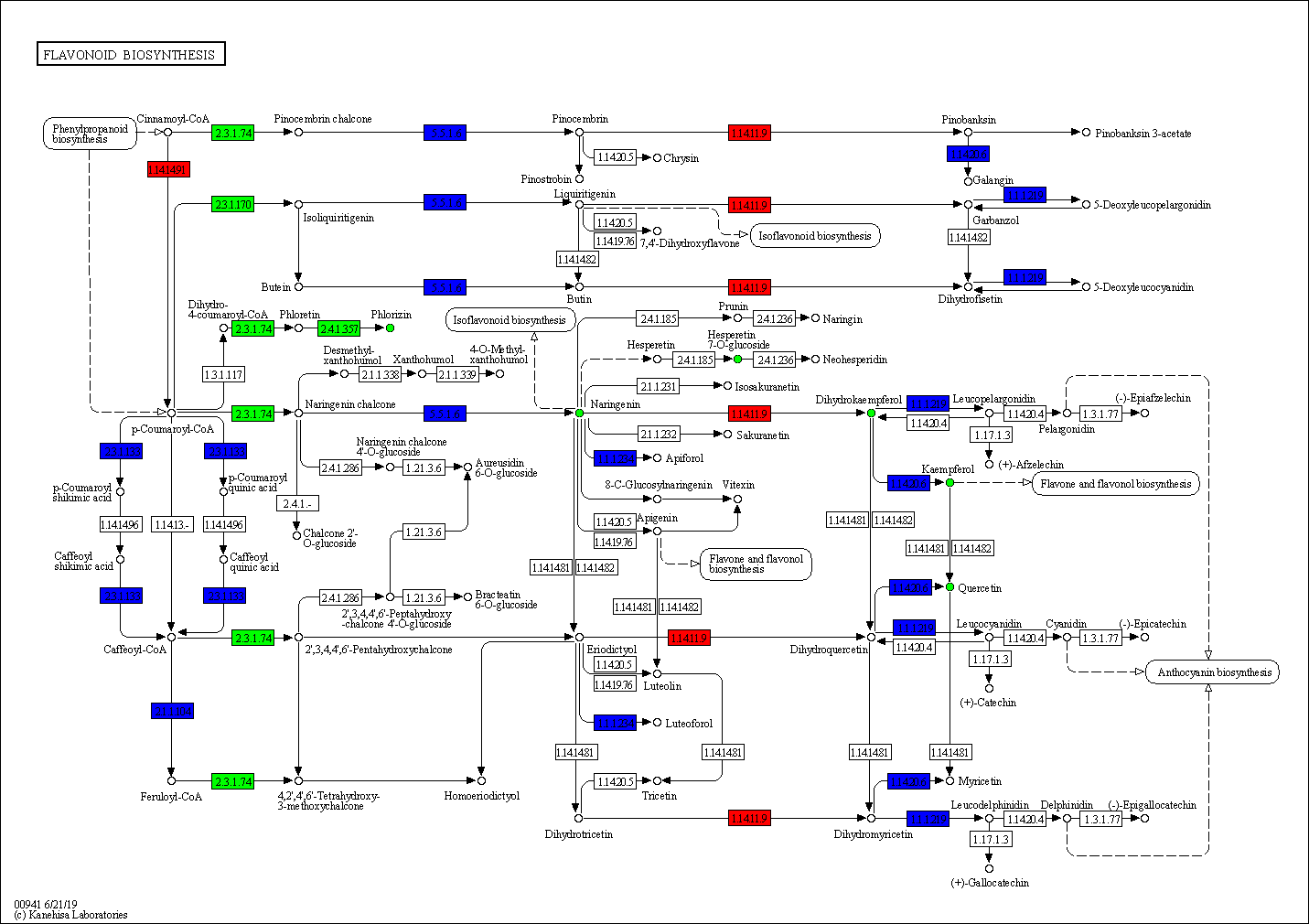


A

B


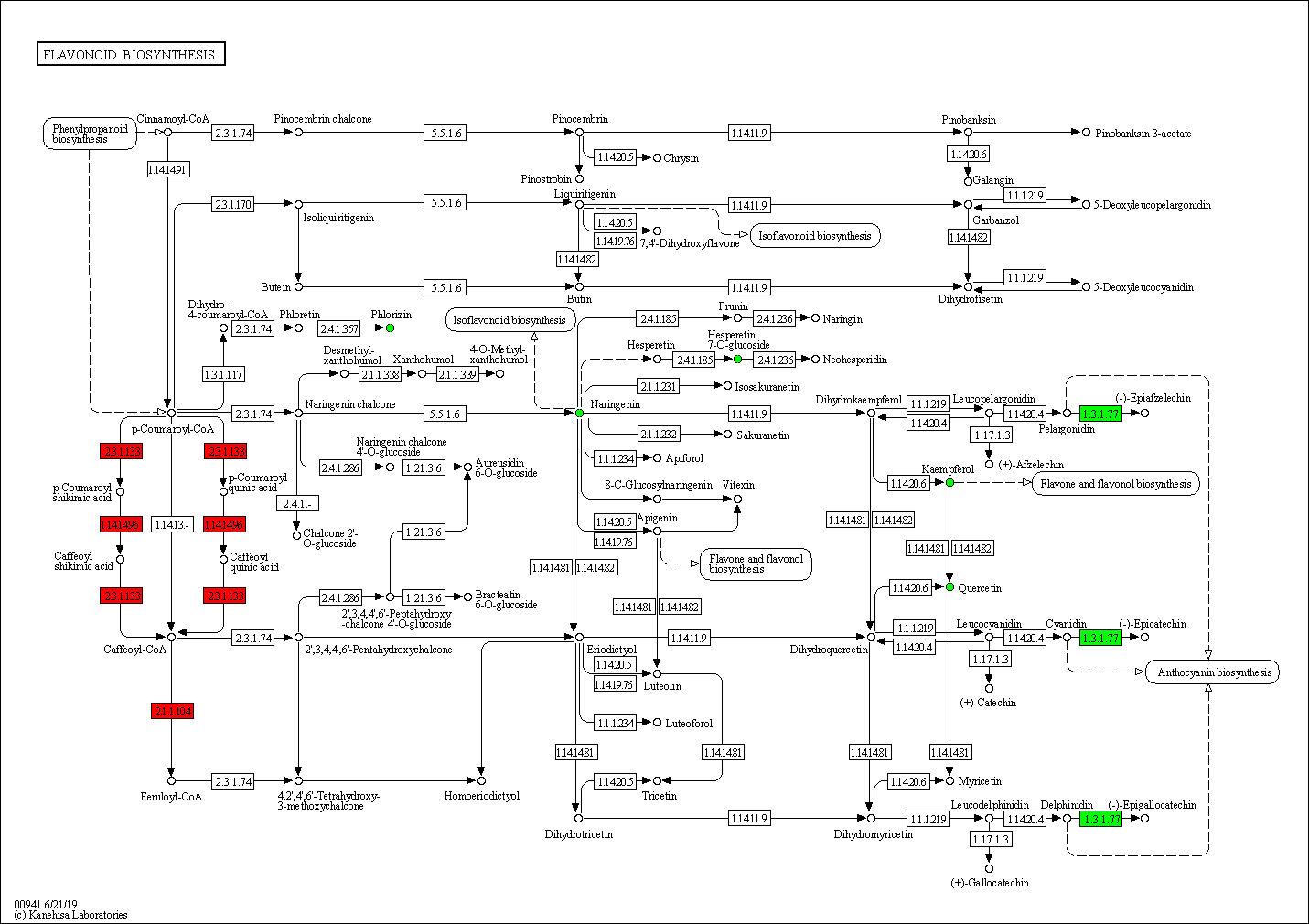


C


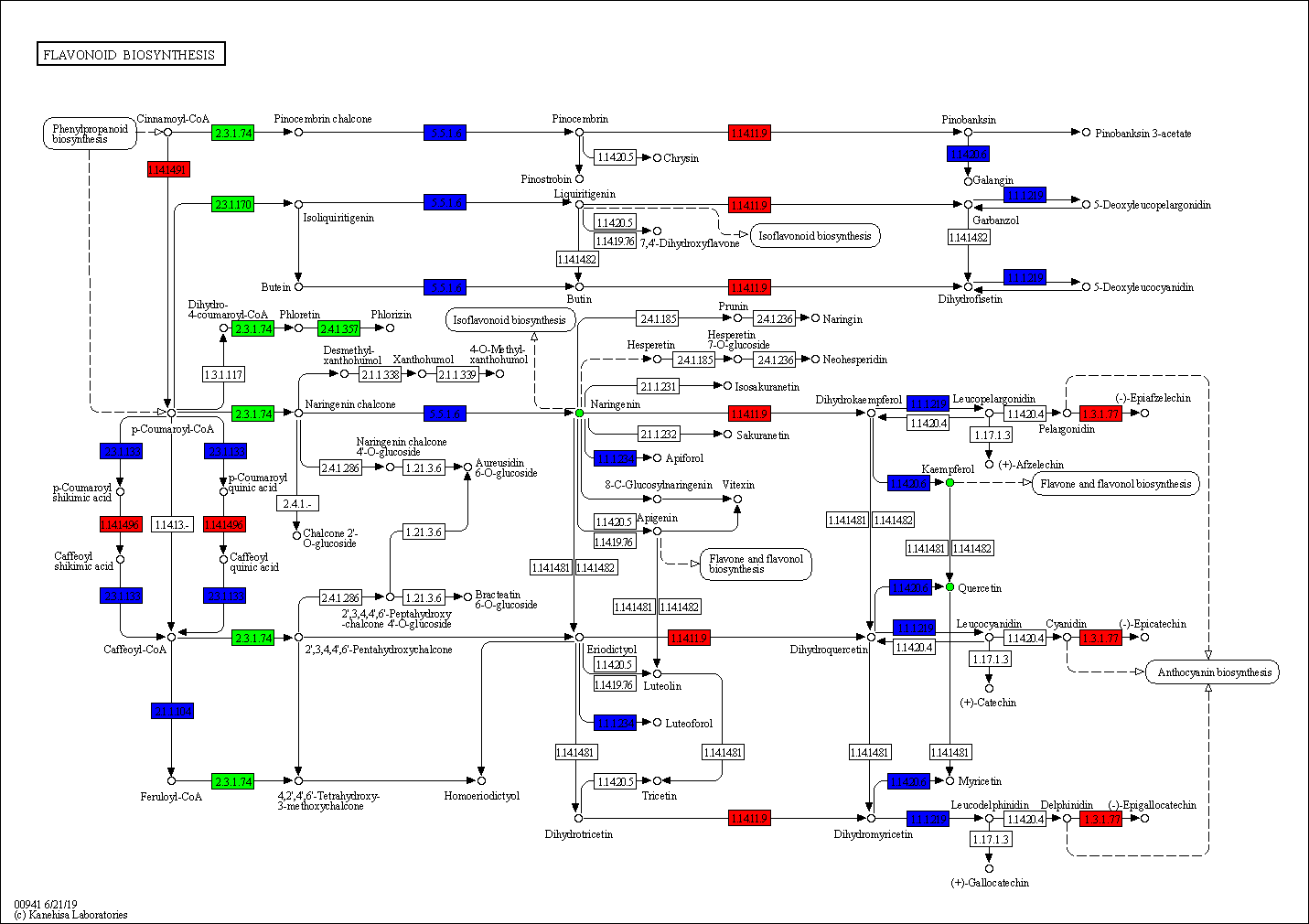


Figure S9. Biosynthesis pathway of flavonoids

Note:A. Drought-Control_vs_Drought; B. Rewater-Control_vs_Rewater; C. Rewater_vs_Drought.

In the figure, red indicates that the gene / metabolite is up-regulated, green indicates that the gene / metabolite is down regulated, and blue indicates that there are both up-regulated and down regulated genes.
