# Supplementary material for: Integrating transcriptomics and metabolomics to analyze quinoa (Chenopodium quinoa Willd.) responses to drought stress and rewatering
Source: Front Plant Sci. 2022 Oct 26;13:988861. doi: 10.3389/fpls.2022.988861 (PMC9645111; doi:10.3389/fpls.2022.988861)
Supplement: Supplementary file 1 [file DataSheet_1.zip › Supplementary materials/Supplementary material 2.docx]

**Experimental procedure for transcriptome sequencing:**

Experimental Procedure

1. RNA quantification and qualification

RNA integrity was assessed using the Fragment Analyzer 5400 (Agilent Technologies, CA, USA).

2. Library preparation for Transcriptome sequencing

Total RNA was used as input material for the RNA sample preparations. Sequencing libraries were generated using NEBNext®UltraTM RNA Library Prep Kit for Illumin®(NEB,USA) following manufacturer's recommendations and index codes were added to attribute sequences to each sample.

Briefly, mRNA was purified from total RNA using poly-T oligo-attached magnetic beads. Fragmentation was carried out using divalent cations under elevated temperature in NEBNext First Strand Synthesis Reaction Buffer (5X). First strand cDNA was synthesized using random hexamer primer and M-MuLV Reverse .

Transcriptase(RNase H). Second strand cDNA synthesis was subsequently

performed using DNA Polymerase I and RNase H. Remaining overhangs were converted into blunt ends via exonuclease/polymerase activities. After adenylation of 3' ends of DNA fragments, NEBNext Adaptor with hairpin loop structure were ligated to prepare for hybridization. In order to select cDNA fragments of preferentially 250~ 300 bp in length, the library fragments were purified with AMPure XP system (Beckman Coulter, Beverly, USA). Then 3µL USER Enzyme (NEB, USA) was used with sizeselected,adaptor-ligated cDNA at 37°C for 15 min followed by 5 min at 95°C before PCR. Then PCR was performed with Phusion High-Fidelity DNA polymerase, Universal PCR primers and Index (X) Primer. At last, PCR products were purified (AMPure XP system) and library quality was assessed on the Agilent Bioanalyzer 2100 system.

3. Clustering and sequencing

The clustering of the index-coded samples was performed on a cBot Cluster

Generation System using TruSeq PE Cluster Kit v3-cBot-HS (Illumia) according to the manufacturer's instructions. After cluster generation, the library preparations were

sequenced on an Illumina Novaseq 6000 platform and 150 bp paired-end reads were

Generated (The sequencing strategy is PE150 , i.e. double end sequencing with 150bp per reads).
